# Supplementary material for: A systematic review and meta-analysis of the relationship between subjective interoception and alexithymia: Implications for construct definitions and measurement
Source: PLoS One. 2024 Nov 7;19(11):e0310411. doi: 10.1371/journal.pone.0310411 (PMC11542822; doi:10.1371/journal.pone.0310411)
Supplement: S2 File — (DOCX) [file pone.0310411.s002.docx]

# **Inter-Rater Reliability**

## **Article Screening**

Database searches employing the pre-registered search strategies identified 232 articles. Following the automated removal of duplicate articles through Covidence (*n* = 97), 135 studies remained and were screened by the three reviewers (KVB, NA, JK) against titles and abstracts. Following title and abstract screening, acceptable agreement amongst reviewers was observed (69 to 74%); however, inter-rater reliability according to Cohen’s kappa was slight to fair (𝜅 = 0.10-0.39), necessitating discussions amongst reviewers to reach consensus. Following these discussions, 103 papers were collectively deemed as not meeting inclusion criteria. Thirty-one papers subsequently remained and were assessed for full-text eligibility. Fifteen studies were excluded and a total of 16 studies identified through database searches were included. Google Scholar searches yielded 797 results; of these, 58 studies were retrieved. Eleven studies identified via Google Scholar were deemed eligible and relevant; included studies then totalled 27. Reference lists for these studies were scanned for additional sources, of which five were identified. These were screened against eligibility criteria and included. We identified that one study was ineligible following screening, as results concerned the same sample [51]. To maintain independence of observations, the study was excluded. Overall, full-text screening agreement was fair to acceptable, requiring further discussion to resolve inconsistencies in screening (67 to 89% agreement; Cohen’s 𝜅 = 0.27 to 0.46). Upon resolution of conflicts between the reviewers through discussions, the number of articles included in final reporting was 32.

## **Data Extraction**

To assess the reliability of the coding process, we calculated the percentage agreement among three independent coders (KVB, NG, and JK). The analysis involved 32 articles, each coded by the three raters. The primary metric for evaluating agreement was the percentage of articles for which all three raters agreed, with a focus on the number of errors made by each coder. Each article was independently reviewed and coded by the three raters. An error was recorded when a coder's extraction differed from the consensus of the other two coders. Out of the 32 articles, the number of articles where each coder made errors (i.e., differed from the consensus) was as follows: KVB: 3 errors, NG: 4 errors, JK: 5 errors. The percentage agreement was calculated based on the number of articles without errors for each coder. Specifically, this involved a calculation of subtracting the number of errors made by each coder from the total number of articles coded. This result was then divided by the total number of articles and multiplied by 100 to obtain a percentage. The percentage agreement among the three coders was relatively high, ranging from 84.4 to 90.6%, indicating a good level of reliability in the coding process. All errors were resolved following further review of the article and discussions.

## **Risk of Bias**

To ensure the reliability of the risk of bias assessment, we assessed the intra-class correlation (ICC) for the overall risk of bias indicated in each article provided by the three reviewers (e.g., overall low risk). A two-way random effects model with consistency type was used to account for variability amongst both the reviewers and the articles. The ICC for single measures was 0.60 (95% CI: 0.41–0.76), indicating good reliability. The ICC for average measures was 0.82 (95% CI: 0.67–0.91), indicating excellent reliability. Together, these values suggested a high level of agreement among the reviewers regarding the risk of bias present amongst the included articles.
