# Supplementary material for: A systematic review and meta-analysis of the relationship between subjective interoception and alexithymia: Implications for construct definitions and measurement
Source: PLoS One. 2024 Nov 7;19(11):e0310411. doi: 10.1371/journal.pone.0310411 (PMC11542822; doi:10.1371/journal.pone.0310411)
Supplement: S3 File — (DOCX) [file pone.0310411.s003.docx]

## **Risk of bias assessment**

Quality ratings varied across studies. Most studies were of moderate quality, with a high level of agreement among the reviewers (see File S2 of Supporting Information). The main sources of bias in the included studies were lacking sample size determinations, unclear or undisclosed eligibility criteria, poor descriptions of validity and reliability of administered measures, unjustified scoring methods, incomplete reasons for missing data, incomplete or omitted measures of variability for outcome measures, and a lack of reporting on statistical assumptions. The below tables provide risk of bias assessments for each included article, according to the 22 STROBE checklist components.

**Table S1. Risk of bias assessment based on STROBE checklist criteria– Introduction and Methods.**

|  | Introduction | | |  | Methods | | | | | | | | |
| --- | --- | --- | --- | --- | --- | --- | --- | --- | --- | --- | --- | --- | --- |
| Study | Title and Abstract | Background and Rationale | Objectives |  | Study Design | Setting | Participants | Variables | Data/ Measurement | Bias | Study Size | Quantitative Variables | Statistical Methods |
| Berenguer et al. (2019) | Low | Low | Low |  | Medium | Medium | Low | Low | Medium | Low | High | Medium | Medium |
| Betka et al. (2018) | Medium | Low | Low |  | Low | Medium | Medium | Low | Low | Low | High | Low | Low |
| Bonete et al. (2023) | Low | Low | Low |  | Low | Medium | Medium | Low | Low | Low | Low | Low | Low |
| Brand et al. (2022) | Low | Medium | Medium |  | Low | Medium | Medium | Low | Low | Low | Medium | Low | Medium |
| Brewer et al. (2016) | Low | Low | Low |  | Medium | High | Low | Medium | Medium | Medium | High | Medium | Medium |
| Campos et al. (2021) | Low | Low | Low |  | Low | Low | Medium | Low | Low | Low | Low | Low | Low |
| Da Costa Silva et al. (2022) | Low | Low | Low |  | Low | Low | Medium | Low | Low | Low | High | Low | Low |
| Desdentado et al. (2022) | Low | Low | Low |  | Low | Low | Low | Low | Low | Low | High | Low | Low |
| Dunn et al. (2022) | Low | Low | Low |  | Low | Medium | Medium | Low | Medium | Low | High | Medium | Medium |
| Edwards and Lowe (2021) | Low | Low | Low |  | Low | Low | Medium | Low | Low | Low | High | Low | Low |
| Ernst et al. (2014) | Low | Low | Low |  | Low | Medium | Medium | Low | Low | Medium | High | Low | Low |
| Ferraro and Taylor (2021) | Low | Low | Low |  | Low | Low | Low | Low | Low | Medium | High | Low | Low |
| Fiene et al. (2018) | Medium | Low | Low |  | Low | Low | Low | Low | Medium | Low | High | Low | High |
| Gaggero et al. (2021) | Low | Low | Low |  | Low | High | Medium | Low | Low | Low | Medium | Low | Low |
| Hassen et al. (2023) | Low | Low | Low |  | Low | Low | Low | Low | Low | Low | High | Low | Low |
| Huang et al. (2022) | Low | Low | Low |  | Low | Low | Medium | Low | Low | Low | High | Low | Low |
| Jakobson et al. (2021) | Low | Low | Low |  | Low | High | Medium | Low | Low | Medium | High | Low | Low |
| Longarzo et al. (2015) | Low | Low | Low |  | Low | Medium | Medium | Low | Low | Medium | Low | Medium | Low |
| Lyvers and Thorberg (2023) | Low | Low | Low |  | Low | Low | Medium | Low | Low | Low | High | Low | Low |
| Morales et al. (2022) | Medium | Low | Low |  | Low | Low | Medium | Low | Low | Medium | High | Low | Low |
| Mul et al. (2018) | Low | Low | Low |  | Low | Medium | Medium | Low | Low | Low | High | Low | Medium |
| Murphy et al. (2020) | Medium | Low | Low |  | Low | Low | Medium | Low | Medium | Low | High | Medium | Low |
| Pink et al. (2021) | Low | Low | Low |  | Low | Low | Medium | Low | Low | Low | High | Low | Low |
| Riccardi et al. (2021) | Low | Low | Low |  | Low | Low | Medium | Low | Medium | Low | Low | Low | Low |
| Schmitz et al. (2021) | Low | Low | Low |  | Low | Low | Medium | Low | Low | Low | Low | Low | Low |
| Sweetnam et al. (2023) | Low | Low | Low |  | Low | Low | Medium | Low | Low | Low | Low | Low | Low |
| Taylor et al. (1996) | Low | Low | Low |  | Low | Low | Medium | Low | Low | Low | Low | Low | Low |
| Tünte et al. (2022) | Low | Low | Low |  | Low | Low | Medium | Low | Medium | Medium | High | Low | Low |
| Ventura-Bort et al. (2021) | Low | Low | Low |  | Low | Low | Medium | Low | Low | Low | Low | Low | Low |
| Vinni et al. (2023) | Low | Low | Low |  | Low | Medium | Medium | Low | Low | Low | Low | Low | Low |
| Vlemincx et al. (2021) | Low | Low | Low |  | Low | Low | Low | Low | Low | Low | High | Low | Low |
| Zahid et al. (2023) | Medium | Low | Low |  | Low | Low | Medium | Low | Low | Low | High | Low | Low |
| Zamariola et al. (2018) | Low | Low | Low |  | Low | Medium | Low | Low | Low | Low | High | Low | Medium |

**Table S2. Risk of Bias assessment based on STROBE checklist criteria – Results, Discussion, and Funding.**

|  | Results | | | | |  | Discussion | | | |  | Other |
| --- | --- | --- | --- | --- | --- | --- | --- | --- | --- | --- | --- | --- |
| Study | Participants | Descriptive  Data | Outcome  Data | Main  Results | Other  Analyses |  | Key  Results | Limitations | Interpretation | Generalisability |  | Funding |
| Berenguer et al. (2019) | Medium | High | Low | Low | Low |  | Low | Medium | Low | Low |  | Low |
| Betka et al. (2018) | Low | Low | Low | Low | Low |  | Low | Low | Low | Medium |  | Low |
| Bonete et al. (2023) | Low | Low | Low | Low | Low |  | Low | Low | Low | Low |  | Low |
| Brand et al. (2022) | Low | Low | Low | Low | Low |  | Low | Low | Low | Low |  | Low |
| Brewer et al. (2016) | Low | Low | Medium | Medium | Low |  | Low | Low | Low | Low |  | Low |
| Campos et al. (2021) | Low | Low | Low | Low | Low |  | Low | Low | Low | Low |  | Low |
| Da Costa Silva et al. (2022) | Low | Medium | Low | Low | Low |  | Low | Low | Low | Low |  | High |
| Desdentado et al. (2022) | Low | Low | Low | Low | Low |  | Low | Low | Low | Low |  | Low |
| Dunn et al. (2022) | Low | Medium | Medium | Low | Low |  | Low | Medium | Low | Low |  | Low |
| Edwards and Lowe (2021) | Low | Low | Low | Low | Low |  | Low | Low | Low | Low |  | Low |
| Ernst et al. (2014) | Medium | Medium | Low | Medium | Low |  | Low | Low | Low | Low |  | Low |
| Ferraro and Taylor (2021) | Low | Low | Low | Low | Low |  | Low | Low | Low | Low |  | Low |
| Fiene et al. (2018) | Medium | Low | Medium | Low | Medium |  | Low | Low | Low | Low |  | Low |
| Gaggero et al. (2021) | Medium | Low | Low | Low | Low |  | Low | Low | Low | Low |  | Medium |
| Hassen et al. (2023) | Low | Low | Low | Low | Low |  | Low | Low | Low | Medium |  | Low |
| Huang et al. (2022) | Low | Low | Low | Low | Low |  | Low | Medium | Low | Low |  | Low |
| Jakobson et al. (2021) | Low | Medium | Low | Low | Low |  | Low | Medium | Low | Low |  | Low |
| Longarzo et al. (2015) | Low | Low | Low | Low | Medium |  | Low | Medium | Low | Low |  | Low |
| Lyvers and Thorberg (2023) | Low | High | Low | Low | Low |  | Low | Low | Low | Low |  | Low |
| Morales et al. (2022) | Low | Low | Low | Low | Low |  | Low | Low | Low | Low |  | Low |
| Mul et al. (2018) | Medium | Low | Low | Low | Low |  | Low | Low | Low | Low |  | Low |
| Murphy et al. (2020) | Low | Low | Medium | Low | Low |  | Low | Low | Low | Low |  | Low |
| Pink et al. (2021) | Low | Low | Low | Low | Medium |  | Low | Low | Low | Low |  | Low |
| Riccardi et al. (2021) | Low | Low | Low | Low | Low |  | Low | Low | Low | Low |  | High |
| Schmitz et al. (2021) | Low | Low | Low | Low | Low |  | Low | Low | Low | Low |  | Low |
| Sweetnam et al. (2023) | Low | Low | Low | Low | Low |  | Low | Low | Low | Low |  | Low |
| Taylor et al. (1996) | Low | Low | Medium | Low | Low |  | Low | Low | Low | Low |  | Low |
| Tünte et al. (2022) | Low | Low | Low | Low | Low |  | Low | Low | Low | Low |  | High |
| Ventura-Bort et al. (2021) | Low | Low | Medium | Low | Medium |  | Low | Low | Low | Low |  | High |
| Vinni et al. (2023) | Low | Low | Low | Low | Low |  | Low | Low | Low | Low |  | High |
| Vlemincx et al. (2021) | Low | Low | Low | Low | Low |  | Low | Low | Low | Low |  | Low |
| Zahid et al. (2023) | Low | Low | Low | Low | Low |  | Low | Low | Low | Low |  | Low |
| Zamariola et al. (2018) | Low | Low | Low | Low | Medium |  | Low | Medium | Low | Low |  | Low |
