# Supplementary material for: A systematic review and meta-analysis of the relationship between subjective interoception and alexithymia: Implications for construct definitions and measurement
Source: PLoS One. 2024 Nov 7;19(11):e0310411. doi: 10.1371/journal.pone.0310411 (PMC11542822; doi:10.1371/journal.pone.0310411)
Supplement: S4 File — (DOCX) [file pone.0310411.s004.docx]

| **Table S3. Characteristics of included studies.** | | | | | | | | | | | | | | | |
| --- | --- | --- | --- | --- | --- | --- | --- | --- | --- | --- | --- | --- | --- | --- | --- |
| Study | Country | Study Design | Sample Characteristics | *N* | *M*Age | % Female | Clinical  Condition | Eligibility  Criteria | Interoceptive  Construct | Interoceptive  Scale | Analysed Interoceptive Scales | Alexithymia  Scale | Analysed Alexithymia Scales | Covariates | Results |
| Berenguer et al. (2023) | Portugal | Cross-sectional | Non-clinical males and females | 340 | F: 24.6 M: 26.2 | 67% | Non-clinical | Exclusion: antidepressant use; health conditions interfering with sexual function; aged ≤17 | Interoceptive Awareness | MAIA | MAIA-Total | TAS-20 | DIF DDF EOT TAS-Total | None | In total sample, MAIA total and TAS scales significantly negatively correlated; small for EOT, and moderate for TAS-Total, DIF, DDF.  In female sample, MAIA total and TAS scales significantly negatively correlated; small for EOT, and moderate for TAS-Total, DIF, DDF. In male sample, small for DIF, DDF, EOT, moderate for TAS-Total. |
| Ben Hassen et al. (2023) | Spain | Cross-sectional | Adults with technical academic training, non-technical academic training, and adults with ASD | Total: 77 Tech: 30 Non-Tech 20 ASD: 27 | Tech: 23.5 Non-Tech: 22.4 ASD: 32.5 | Technical: 46.7% Non-Technical: 85% ASD: 50% | ASD | Diagnosis confirmed via interview; family interview; results on 4 standardised psychopathological tests. Technical and Non-Technical: Not reported. | Interoception | BPQ | BPQ-SF-Total | TAS-20 | DIF DDF EOT TAS-Total | Age, gender | In ASD group, negative correlations between BPQ-SF and TAS scales, medium (DIF, DDF) and large (TAS-Total) in magnitude. EOT n.s.. No significant correlations between TAS scores and BPQ-SF in technical and non-technical academic training groups. |
| Betka et al. (2018) | UK | Cross-sectional | Students and staff | 590 |  | 74% | Non-clinical | Not reported. | Interoceptive Sensibility | BPQ | BPQ-BA | TAS-20 | TAS-Total | Age, gender, education | Small positive correlations between DIF, DDF, and TAS-Total and BPQ-BA. EOT ns. |
| Bonete et al. (2023) | Spain | Cross-sectional | Men diagnosed with ASD aged 21-58; men with neurotypical development, aged 18-58 | 60 ASD: 33 CG: 35 | ASD: 34.3 CG: 33.4 | 0% | ASD | Inclusion: ASD group: aged 18+, confirmed ASD diagnosis, language proficiency, score above 6 on AQ-10. CG: aged 18+, no ASD or other clinical diagnosis, score below 6 on AQ-10. | Interoceptive Confusion | ISQ | ISQ-Total | TAS-20 | TAS-Total | None | Large positive correlations between ISQ Total and TAS-Total for ASD and HC groups; stronger magnitude in HCs. |
| Brand et al. (2022) | Austria, Germany | Cross-sectional | German speaking adults drawn from general population; German and Austrian university students | Total: 3462 Sample 1: 484  Sample 2: 1509  Sample 3: 388  Sample 4: 77 Sample 5: 226  Sample 6: 254  Sample 7: 522 | Sample 1: Mage=27.8 years, Sample 2: Mage=33.3 years, Sample 3: Mage=31.0 years, Sample 4:, Mage=23.5 years, Sample 5: Mage=22.8 years, Sample 6: Mage=24.5 years , Sample 7: Mage=23.4 years | Sample 1: 71.2% Sample 2: 79.5% Sample 3: 55.7% Sample 4: 42.8% Sample 5: 72.2% Sample 6: 83.2% Sample 7: 81.1% | Unclear | Exclusion: no report of high proficiency German level; underaged; left items unanswered, and/or responded too fast or slow; reported neurological or heart disease. | Self-Reported Interoceptive Accuracy | IAS | IAS-Total | TAS-20 | TAS-Total | None | Medium negative correlation between IAS and TAS-Total in Potsdam; small negative for Vienna IAS. |
| Brewer et al. (2016) | UK | Cross-sectional | Unclear. | 653 | Not reported. | Not reported | Not reported. | Not reported. | Interoceptive Sensibility | ICQ | ICQ-Total | TAS-20 | TAS-Total | None | Strong positive correlation between ICQ and TAS-Total. |
| Campos et al. (2021) | Portugal | Cross-sectional | Community sample | 515 | 30.7 | 60% | Non-clinical | Not reported. | Self-Reported Interoceptive Accuracy | IAS | IAS-Total | TAS-20 | TAS-Total | Not reported. | Significant correlations between TAS-Total and interoceptive self-reports, small negative (IAS) and medium positive (BPQ-R) in magnitude. BPQ-BA n.s.. |
|  |  |  |  |  |  |  |  |  | Self-reported Interoceptive Attention | BPQ | BPQ-BA |  |  |  |  |
|  |  |  |  |  |  |  |  |  |  |  | BPQ-R |  |  |  |  |
| Da Costa Silva et al. (2022) | France | Cross-sectional | French adults | 308 | 35.2 | 61.40% | Non-clinical | Inclusion: No history of neuropsychiatric disease and chronic pain; aged 18-65; able to read and understand French. | Interoceptive Awareness | MAIA-2 | Not., ND, NW, AR, EA, SR, BL, Trust. | TAS-20 | DIF DDF EOT TAS-Total | Not reported. | Negative correlations between DIF and MAIA scales, small (Not., ND, NW, EA, SR) and medium (MAIA-Total, AR, BL, Trust.) in magnitude. Negative correlations between DDF and MAIA scales, small (Not., ND, AR, EA, SR, BL, Trust.) and medium (MAIA-Total) in magnitude. NW ns. Negative correlations between EOT and MAIA scales, small (ND, NW, Trust.), medium (Not., AR, EA, SR, BL) and large (MAIA-Total) in magnitude. Negative correlations between TAS-Total and MAIA scales, small (ND), medium (Not., AR, EA, SR, BL, Trust.), and large (MAIA-Total) in magnitude. NW ns. |
| Desdentado et al. (2022) | Spain | Cross-sectional | Healthy native Spanish adults | 391 | 29 | 61% | Non-clinical | Exclusion: history of neurological disease or psychiatric disorders; taking psychotropic drugs; not native Spanish speaker. | Interoceptive Sensibility | MAIA-2 | Not., ND, NW, AR, EA, SR, BL, Trust. | TAS-20 | DIF DDF EOT TAS-Total | Not reported. | Negative correlations between DIF and MAIA scales, small (Not., ND, NW, AR, SR, BL, Trust.) in magnitude. EA ns. Negative correlations between DDF and MAIA scales, small (ND, NW, AR, EA, SR, BL, Trust.) in magnitude. Not. ns. Negative correlations between EOT and MAIA scales, small (Not., AR, EA, SR, BL, Trust.) in magnitude. ND and NW ns. Negative correlations between TAS-Total and MAIA scales, small (ND, NW, AR, EA, SR, BL, Trust.) in magnitude. Not. ns. MAIA ND, NW, AR, and Trust. scales significantly negatively predicted alexithymia in structural equation models. Not., EA, SR, BL n.s. |
| Dunn et al. (2022) | US | Cross-sectional | University students | 74 | 26 | 90% | Non-clinical | Not reported. | Interoceptive Impact | SPI | Registration, Avoiding, Sensitivity, Seeking | PAQ | PAQ-Total | None | Small positive correlation between PAQ-Total and SPI Registration (lack of awareness of interoceptive input) *r*=.260.  SPI Avoiding, SPI Sensitivity, SPI Seeking n.s. |
| Edwards and Lowe (2021) | UK | Cross-sectional | Adults with possible alexithymia | 242 |  | 51% | Non-clinical | Inclusion: Aged 18+; good ability to read English; normal to corrected to normal vision; internet access; reported difficulty in labeling and describing emotions, which they believed was because of alexithymia. | Interoceptive Awareness | MAIA-2 | Not., ND, NW, AR, EA, SR, BL, Trust. | TAS-20 | DIF DDF EOT TAS-Total | None | Negative correlations between DIF and MAIA scales, small (Not., ND, AR, EA, BL) in magnitude. NW, SR, Trust. ns. Negative correlations between DDF and MAIA scales, small (ND, AR, SRBL) and medium (NW, Trust.) in magnitude. Not., EA ns. Positive correlations between EOT and MAIA scales, small (Not.)and medium (AR, EA, SR, BL, Trust.) in magnitude. ND and NW ns. Negative correlations between TAS-Total and MAIA scales, small (ND, AR) and moderate (Trust.)in magnitude. Not., NW, EA, SR, BL n.s.. Stepwise linear regression models showed TAS-Total negatively predicted by ND, AR, and EA. SR positively predicted TAS-Total. DIF negatively predicted by ND and AR. DDF negatively predicted by ND and AR. EOT negatively predicted by EA. |
| Ernst et al. (2014) | Switzerland | Cross-sectional | Healthy adults | 18 | 27.1 | 59% | Non-clinical | Exclusion: Major medical illnesses; histories of seizures; head trauma with loss of consciousness and pregnancy; any psychiatric or neurologic disorder; history of substance dependence. | Interoceptive Awareness | BPQ | BPQ-BA | TAS-20 | TAS-Total | None | Strong positive correlations between BPQ scales and TAS total: BPQ-Total, BPQ-BA, BPQ-SR, BPQ-AR and BPQ-SS. |
|  |  |  |  |  |  |  |  |  |  |  | BPQ-SS |  |  |  |  |
|  |  |  |  |  |  |  |  |  |  |  | BPQ-SR |  |  |  |  |
|  |  |  |  |  |  |  |  |  |  |  | BPQ |  |  |  |  |
|  |  |  |  |  |  |  |  |  |  |  | BPQ-AR |  |  |  |  |
| Ferraro and Taylor (2021) | Australia | Cross-sectional | Australian adults drawn from a community sample and university students | 269 |  | 22% | Non-clinical | Not reported. | Interoceptive Awareness | MAIA-2 | MAIA-Total | TAS-20 | TAS-Total | None | Medium positive correlation between MAIA Total and TAS-Total). MAIA Total negatively predicted TAS-20 in serial mediation model. |
| Fiene et al. (2018) | Australia | Cross-sectional | Adults with and without ASD from universities and the general population | 511 Autism: 52 Neurotypical: 459 | ASD: 35.5 Typical: 33.5 | Autistic: 51.9% Neurotypical: 62.3% | ASD | ASD: Previously diagnosed with autism by a qualified professional (paediatrician, psychiatrist, clinical psychologist). Neurotypical: Not reported. | Interoceptive challenges | ISQ | ISQ-Total | TAS-20 | TAS-Total | None | Large positive correlation between ISQ and TAS-Total. |
| Gaggero et al. (2021) | US, Singapore, Italy | Cross-sectional | IT: adults aged 18-53, primarily University students Sing: undergraduate students aged 18-28 US: adults aged 22-58 | Total: 814 Italy: 325 Singapore: 239 US: 250 | IT: 23.5 | Italy: 68% Singapore: 62.8% US: 48% | Non-clinical | Italian: Italian native speakers; aged 18-35 years old; university students or educational level at least equivalent to Italian Bachelor’s degree. Singapore: Not reported. US: aged 25-30, US bachelor’s degree as minimum educational level, US as country of residence. | Subjective Interoception | BPQ | BPQ-Total, BPQ-R, BPQ-R-Supra, BPQ-R-Sub | TAS-20, BVAQ | DIF DDF EOT TAS-Total | None | Negative correlations between TAS-Total and interoceptive self-reports, small (Not., ND, NW, ND, AR, EA, SR), medium (IAS, MAIA-Total, BL, Trust.) in magnitude. Positive correlations small (BPQ-AR) and medium (ICQ). BPQ-BA n.s.. Negative correlations between DIF and interoceptive self-reports, small (Not., ND, AR, SR, BL), medium (IAS, MAIA-Total, NW, Trust.), and large (ICQ) in magnitude. Positive medium correlation with BPQ-AR. BPQ-BA, EA n.s.. Negative correlations between DDF and interoceptive self-reports, small (IAS, BPQ-AR, Not., ND, AR, EA, SR, BL, Trust.) in magnitude. Positive correlations, small (BPQ-AR) and medium (ICQ) in magnitude. BPQ-BA, ND n.s.. Negative correlations between EOT and interoceptive self-reports, small (BPQ-BA, MAIA-Total, Not., AR, EA, SR, BL, Trust.) in magnitude. Positive small correlation with ICQ. IAS, ND, NW n.s.. Negative correlations between BVAQ-Total and interoceptive self-reports, small (IAS, Not., ND, AR, EA, SR, Trust.) and medium (BL) in magnitude. Positive correlations, small (BPQ-BA, BPQ-AR) and medium (ICQ) in magnitude. NW n.s.. Machine Learning models estimated that Alexithymia (TAS-20) was best predicted by ICQ, MAIA-Not-Worrying, MAIA-Attention Regulation, and MAIA-Noticing. BVAQ-Cognitive Alexithymia was best predicted by ICQ, MAIA-Emotional Awareness, MAIA-Trusting, MAIA-Body Listening, and BPQ-Reactivity. BVAQ-Affective Alexithymia was best predicted by MAIA-Body Listening, MAIA-Not-Worrying, MAIA-Not-Distracting, and BPQ-Awareness. Negative correlations between BVAQ-C and interoceptive self-reports, small (BPQ-BA, BPQ-AR, Not., ND, EA, SR) and medium (IAS, MAIA-Total, BL, Trust.) in magnitude. Large positive correlation with ICQ. NW n.s.. |
|  |  |  |  |  | Sing: 21.8 |  |  |  |  |  | BPQ-BA |  | BVAQ-Total, BVAQ-A BVAQ-C |  |  |
|  |  |  |  |  | US: 29.5 |  |  |  |  | IAS | IAS-Total |  |  |  |  |
|  |  |  |  |  |  |  |  |  |  | ICQ | ICQ-Total |  |  |  |  |
|  |  |  |  |  |  |  |  |  |  | MAIA-2 | Not., ND, NW, AR, EA, SR, BL, Trust., MAIA-Total |  |  |  |  |
| Huang et al. (2022) | Taiwan | Cross-sectional | Healthy adults aged 20-64 | 224 | 22.1 | 70.10% | Non-clinical | Aged 20-64; native speakers of traditional Chinese; normal vision (with or without correction); no diagnosis of mental disorders that influence reality testing or cognitive ability (e.g., schizophrenia, dementia) | Interoceptive Sensibility | MAIA | Not., ND, NW, AR, EA, SR, BL, Trust. | TAS-20 | TAS-Total | Social desirability | Negative correlations between TAS-Total and MAIA scales, small (NW, AR, SR, BL, Trust.) in magnitude. Not., EA n.s.  Cluster analysis produced low, moderate, and high IS groups based on MAIA scale scores. Low IS, Moderate IS > High IS on TAS-Total, DDF, EOT. DIF n.s.. |
| Jakobson et al. (2021) | Canada | Cross-sectional | University students enrolled in psychology | 201 | 19.7 | 55.70% | Non-clinical | Not reported. | Self-Reported Interoceptive Accuracy | IAS | IAS-Total | TAS-20 | TAS-Total | Age | Small negative correlations between IAS and TAS-Total, DIF, DDF, EOT. IAS scores negatively predicted TAS-Total. |
| Longarzo et al. (2015) | Italy | Cross-sectional | Healthy university students and staff from psychology and political science departments | 250 | 27.9 | 70% | Non-clinical | No current or past history of alcohol or drug abuse; no current or past history of major psychiatric diseases; no history of brain injury, stroke, or any other major clinical condition; no past or current use of psychoactive medications. | Interoceptive Awareness | SAQ | SAQ-Total | TAS-20 | DIF DDF EOT TAS-Total | None | Positive correlations between SAQ and TAS scales, small (DDF) and medium (DIF) in magnitude. EOT n.s.. SAQ positively predicted TAS-20. |
| Lyvers and Thorberg (2023) | Australia | Cross-sectional | Young adult alcohol users aged 18-30 | 224 | 24.8 | 66% | Non-clinical | Aged 18-30; no current medication for neurological or psychological disorder, or history of traumatic brain injury. | Interoceptive Sensibility | MAIA-2 | MAIA-Total | TAS-20 | TAS-Total | None | No significant correlation between MAIA-Total and TAS-Total. Correlations between MAIA scales and TAS-Total, small positive (ND) and negative (AR, Trust) TAS-Total significantly negatively predicted MAIA-Total in mediation model predicting alcohol use. |
| Morales et al. (2022) | US | Randomised experimental | Female undergraduate students aged 18 to 29, normal BMI | 128 | 19.3 | 100% | Non-clinical | Inclusion: BMI ≥17.5; history or no history of loss of control eating; English proficiency. Exclusion: ED diagnosis or behaviour; active psychotic symptoms; current suicidal ideation. | Interoceptive Awareness | MAIA | MAIA-Total | TAS-20 | TAS-Total | None | Small negative correlation between TAS-Total and MAIA-Total following experimental manipulation of positive or negative mood. |
| Mul et al. (2018) | UK | Cross-sectional | Adults with and without ASD | 52 ASD: 26 HC: 26 |  | 26.9% | ASD | ASD: Previous ASD diagnosis confirmed via clinical interview.  HCs: Not reported. | Interoceptive Awareness | MAIA | AR, Active and Reactive Strategies (ND, NW, SR, BL), Awareness (Not., EA, Trust.) | TAS-20 | DIF DDF EOT TAS-Total | BMI | Negative partial correlations between EOT and MAIA factors, medium (AR, active and reactive strategies [ND, NW, SR, BL]) and large (awareness [Not., EA, Trust]) in magnitude. HCs, ASD/Alexithymia, and ASD/No Alexithymia groups significantly differed in MAIA factors for awareness, active and reactive strategies, and AR MAIA factors: ASD/Alexithymia group < ASD/No Alexithymia, HCs  In pooled sample, TAS-Total negatively predicted by MAIA awareness (b=-2.42), active and reactive strategies (b=-2.88), AR n.s. (b=0.16), R2=.47. |
| Murphy et al. (2020) | UK | Cross-sectional | Convergent Validity (Study 2): Adults aged 18-91 primarily without psychiatric diagnoses Accuracy and Attention (Study 5) Healthy adults aged 20-56 | Study 2: 76 Study 5: 35 | Study 2: 39.3 Study 5: 28.5 | Study 2: 67.1 Study 5: 74.1 | Study 2: Unclear Study 5: Non-clinical | Not reported for Study 2.  Study 5: No current psychiatric diagnosis; English as first language. | Self-Reported Interoceptive Accuracy | BPQ | BPQ-BA | TAS-20 | TAS-Total | Study 2: Self-esteem Study 5: Age, gender, depression, and anxiety | Study 2: Medium negative correlation between IAS and TAS-Total (r=.43), BPQ-A n.s.. After controlling for self-esteem, negative partial correlation between TAS-20 and IAS small in magnitude. BPQ-A n.s.  Study 5: Large negative correlations between TAS-Total and IAS, ICQ. BPQ n.s. Multiple linear regressions controlled for age, gender, depression, and anxiety; TAS-Total significantly negatively predicted IAS, positively predicted ICQ. TAS-Total n.s. where BPQ was outcome. |
|  |  |  |  |  |  |  |  |  | Self-Reported Interoceptive Attention | IAS | IAS-Total |  |  |  |  |
|  |  |  |  |  |  |  |  |  |  | ICQ | ICQ-Total |  |  |  |  |
| Pink et al. (2021) | UK | Cross-sectional | Healthy females | 254 | 23.8 | 100% | Non-clinical | Identifying as female; no current or historical diagnosis of depression, anxiety or eating disorders; English language proficiency. | Interoceptive Sensibility | MAIA | Not., ND, NW, AR, EA, SR, BL, Trust. | TAS-20 | DIF DDF EOT TAS-Total | None | Recorded at baseline, negative correlations between DIF and MAIA scales, small (NW), medium (Trust), and large (AR) in magnitude. Not., ND, EA, SR, BL ns. Small negative correlations between DDF and MAIA scales (AR, EA, Trust.) in magnitude. Not., ND, NW, SR, BL ns. Small negative correlations between EOT and MAIA scales (Not., AR, EA, SR, BL, Trust.). ND and NW ns. Negative correlations between TAS-Total and MAIA scales, small (NW, AR, EA, SR, BL) and medium (Trust) in magnitude. Not.,ND, BL n.s.. |
| Riccardi et al. (2021) | UK | Cross-sectional | Patients with a diagnosis of functional motor disorders; HC matched for age and gender | Functional group: N=22 HC: N=23 Total: 55 |  | 86.4% | Functional motor disorders | FMD: clinically established and documented FMD. HCs: hospital visitor or staff. Exclusion: language difficulties; learning disability; concurrent neurological, cardiologic or medical conditions,; treatment with medications with direct cardiac effects. | Interoceptive Sensibility | BAQ | BAQ-Total | TAS-20 | TAS-Total | None | No significant Spearman correlation between BAQ and TAS-Total in Functional group only or full sample. |
| Schmitz et al. (2021) | Germany | Cross-sectional | Patients with fibromyalgia; HC matched for age and gender, aged 21-77 | 112 FM: 55 HCs: 55 | 53.8 | 83.90% | Fibromyalgia | Fibromyalgia: Confirmed diagnosis via physician based on 2 criteria. Exclusion: Severe physical diseases, psychosis, and addictions. HCs: meeting DSM-IV criteria of pain disorder or somatic stress disorder. | Interoceptive Sensibility | MAIA | Not., ND, NW, AR, EA, SR, BL, Trust. | TAS-20 | TAS-Total | Depression, anxiety | In fibromyalgia group, negative correlations between TAS-Total and MAIA scales, small (Not., ND, EA, BL, Trust.) and medium (NW, AR, SR) in magnitude. In regression model, no MAIA subscale significantly predicted TAS-Total scores above and beyond anxiety and depression.  For healthy controls, negative correlations between TAS-Total and MAIA scales, small (Not., ND, NW, SR), medium (AR, EA, BL), and large (Trust.) in magnitude. In regression model, only MAIA-Not. and MAIA-EA were significant negative predictors of TAS-Total. |
| Sweetnam and Flack (2023) | Australia | Cross-sectional | Adults aged 18-78 | 349 | 43.7 | 86.40% | Non-clinical | Engaged in weekly exercise; aged 18+ | Interoceptive Awareness | MAIA-2 | MAIA-Total | TAS-20 | TAS-Total | None | Large negative correlation between MAIA-Total and TAS-Total. |
| Taylor et al. (1996) | UK | Cross-sectional | Female patients with AN, matched HCs, university students | Total: 312 AN: 48 HC: 30 Students: 234 | AN: 24.7 HCs: 26.8 Students: 21.6 | AN: 100% HC: 100% Students: 50.4% | AN | AN: patients meeting DSM-III-R criteria for AN. HC: no history of eating disorder. | Interoceptive Awareness | EDI | EDI-IAw | TAS-20 | TAS-Total | None | Medium positive correlations between EDI-IAw and TAS-Total in AN and male students. |
| Tünte et al. (2022) | Austria, Germany | Cross-sectional | German speaking adults drawn from general population; German and Austrian university students | Total: 857 Sample 1: 135 Sample 2: 388 Sample 3: 77 Sample 4: 254 | 23.4 - 30.96 | Sample 1: 77.7%  Sample 2: 55.7% Sample 3: 72.7% Sample 4: 81.1% | Non-clinical | Inclusion: Aged 18-70. Exclusion: no report of high proficiency German level, aged ≤17, left items unanswered, and/or responded too fast or slow; reported neurological or heart disease. | Self-Reported Interoceptive Attention | IATS | IATS-Total | TAS-20 | DIF DDF EOT TAS-Total | None | Positive correlations between TAS-Total and interoceptive self-reports, small (IATS, BPQ-R-Sub) and medium (BPQ-R-Supra) in magnitude. Medium negative correlation with IAS. BPQ-BA n.s. Small positive correlations between DDF and interoceptive self-reports (IATS,BPQ-R-Supra, BPQ-R-Sub). Small negative correlation with IAS. BPQ-BA n.s. Positive correlations between DIF and interoceptive self-reports, small (IATS, BPQ-R-Sub) and medium (BPQ-R-Supra) in magnitude. Medium negative correlation with IAS. BPQ-BA n.s. Small negative correlation between EOT and BPQ-BA. IATS, IAS, BPQ-R-Supra, BPQ-R-Sub n.s. |
|  |  |  |  |  |  |  |  |  |  |  |  |  |  |  |  |
|  |  |  |  |  |  |  |  |  |  |  |  |  |  |  |  |
|  |  |  |  |  |  |  |  |  |  |  |  |  |  |  |  |
|  |  |  |  |  |  |  |  |  |  |  |  |  |  |  |  |
| Ventura-Bort et al. (2021) | Germany | Cross-sectional | University students | 157 | 25.9 | 85.99% | None | Inclusoin: German proficiency. Exclusion: History of neurological disorder; undergoing psychological treatment; suffered psychological disorder in last year; ongoing acute or long-term psychiatric treatment. | Interoceptive Sensibility | ICQ | ICQ-Total | TAS-20 | DIF DDF EOT | None | Negative correlations between DIF and interoceptive self-reports, small (EA), medium (Not., AR, BL), and large (IAS, Trust.) in magnitude. Positive large correlation with ICQ. Negative correlations between DDF and interoceptive self-reports, small (IAS, EA) and medium (AR, BL, Trust.) in magnitude. Positive medium correlation with ICQ. Not. n.s. Negative correlations between EOT and interoceptive self-reports, small (IAS, AR, EA, Trust.) and medium (IAS) in magnitude. ICQ, Not. n.s.. PCA produced sensibility factor, consisting of ICQ, IAS, AR, Trust, DIF, DDF, EOT scales. |
|  |  |  |  |  |  |  |  |  |  | IAS | IAS-Total |  |  |  |  |
|  |  |  |  |  |  |  |  |  |  | MAIA-2 | Not., AR, EA, BL |  |  |  |  |
| Vinni et al. (2023) | Greece | Case control, Cross-sectional | Adult patients with IBD | IBD: 57 (Crohn's: 41, UC: 16) |  | CD: 36.8% UC: 62.5% | IBD (CD, UC) | Exclusion: illicit drug use or alcohol abuse during past year; stroke, cancer, cerebrovascular disease; mental retardation, dementia, psychotic or bipolar disorder; mindfulness-based therapy; lack of fluency in the Greek language. | Interoceptive Sensibility | MAIA | Not., ND, NW, AR, EA, SR, BL, Trust. | TAS-20 | DIF DDF EOT TAS-Total | None | In CD group, medium negative correlations between DIF and MAIA scales (NW, AR, SR, BL, Trust.). Medium negative correlations between DDF and MAIA scales (NW, AR, BL, Trust.). No correlations between EOT and MAIA scales. Medium negative correlations between TAS-Total and MAIA scales (NW, AR, SR, BL, Trust.).In UC group, no significant correlations between TAS scales and MAIA scales. |
| Vlemincx et al. (2021) | Netherlands | Cross-sectional | Undergraduate psychology students; Dutch speaking adults | Students: 357 Dutch adults: 399 | Students: 18.3 Adults: 28.9 | Students: 84.3% Dutch adults: 47.3%) | Non-clinical | Aged 18+; proficiency in Dutch | Self-Reported Interoception | THISQ | THISQ-Total, THISQ-CRA,THISQ-CRD, THISQ-GES | TAS-20 | DIF DDF EOT | None | Small positive correlation between DIF and THISQ-CRA. Small positive correlations between THISQ scales and DDF (CRA, GES). Small negative correlations between EOT and THISQ scales (THISQ-Total, CRA, CRD, GES). |
| Zahid et al. (2023) | Canada | Cross-sectional | Adults from English-speaking countries | 759 |  | 50.60% | Non-clinical | English proficiency; from US, Canada, UK, New Zealand, Australia. | Interoceptive Awareness | MAIA-2 | AR, SR, BL | TAS-20, PAQ | TAS-Total PAQ-Total | None | Small negative correlations between TAS-Total and MAIA scales (AR, SR, BL). Small negative correlations between PAQ-Total and MAIA scales (AR, SR, BL). TAS-Total negatively predicted AR, SR and BL. No PAQ subscales predicted MAIA scales. |
| Zamariola et al. (2018) | Belgium | Cross-sectional | French and Dutch university students | 899 | 20.1 to 22.8 | Study 2 (BAQ/Alexithymia): 74.7% Study 3 (BAQ/Alexithymia): 75.2% Study 4 (MAIA/Alexithymia): 65% Study 5 (MAIA/Alexithymia): 69% Study 6 (MAIA/Alexithymia): 86% | Non-clinical | Not reported. | Interoceptive Sensibility | BAQ | BAQ-Total | TAS-20 | DIF DDF EOT TAS-Total | None | Negative correlations between BAQ and TAS scales, small (DDF) and medium (EOT, TAS-Total) in magnitude. DIF n.s.. Negative correlations between DIF and MAIA scales, small (Not., ND) and medium (NW, AR, Trust.) EA, BL ns. Negative correlations between DDF and MAIA scales, small (NW, AR, SR, BL, Trust.) in magnitude. Not., ND, EA ns. Negative correlations between EOT and MAIA scales, small (Not., AR, EA, SR, BL, Trust.) in magnitude. ND and NW ns. Negative correlations between TAS-Total and MAIA scales, small (Not, SR, BL) and medium (NW, AR, Trust.) ND, EA. ns. Small positive correlation between TAS-Total and IAQ-Aw. Regression models showed that BAQ negatively predicted EOT and TAS-total. IAQ—Aw negatively predicted EOT and TAS-Total. |
|  |  |  |  |  |  |  |  |  |  | MAIA | Not., ND, NW, AR, EA, SR, BL, Trust. |  |  |  |  |
|  |  |  |  |  |  |  |  |  |  |  |  |  |  |  |  |
