# Supplementary material for: A systematic review and meta-analysis of the relationship between subjective interoception and alexithymia: Implications for construct definitions and measurement
Source: PLoS One. 2024 Nov 7;19(11):e0310411. doi: 10.1371/journal.pone.0310411 (PMC11542822; doi:10.1371/journal.pone.0310411)
Supplement: S5 File — (DOCX) [file pone.0310411.s005.docx]

| **Table S4. Interoceptive Self-Report Scales Employed in Included Studies, Abbreviations, Subscales, and Descriptions.** | | |
| --- | --- | --- |
| Measure and Subscales | Abbreviation | Scale Description |
| Body Awareness Questionnaire | BAQ | Assesses self-reported attentiveness to normal non-emotive body processes, such as sensitivity to body cycles and rhythms, ability to detect small changes in normal functioning, and ability to anticipate bodily reactions. |
| Body Perception Questionnaire | BPQ | The subjective experiences of the function and reactivity of target organs and structures that are innervated by the autonomic nervous system |
| Short Form | SF | Abbreviated version of BPQ |
| Body Awareness Scale | BPQ-BA | Sensitivity for and awareness of internal bodily functions. |
| Autonomic Reactivity Scale | BPQ-R-Total | A combined measure of both supra- and sub-diaphragmatic symptoms (e.g., shortness of breath cf. digestive problems). |
| Autonomic Reactivity - Supradiaphragmatic | BPQ-R-Supra | Reactivity of symptoms above the diaphragm |
| Autonomic Reactivity - Subdiaphragmatic | BPQ-R-Sub | Reactivity of symptoms below the diaphragm |
| Stress Response | BPQ-SR | Awareness of perceived changes due to stressful situations |
| Stress Style | BPQ-SS | Typical emotional and physiological responses to stress |
| Eating Disorder Inventory | EDI | Multidimensional questionnaire assessing eating-related attitudes and behaviours and other psychological traits associated with anorexia nervosa and bulimia nervosa |
| Interoceptive Awareness Scale | IAw | The ability to discriminate between sensations and feelings, and between the sensations of hunger and satiety |
| Interoceptive Accuracy Scale | IAS | Self-perceived interoceptive accuracy |
| Interoceptive Attention Scale | IATS | Evaluates self-reported attention to interoceptive signals, such as hunger or pain |
| Interoceptive Confusion Questionnaire | ICQ | Self-perceived trait interoceptive accuracy; assesses the degree to which individuals feel that they struggle to interpret their own non-affective interoceptive states |
| Interoception Sensory Questionnaire | ISQ | Confusion about interoceptive bodily states unless these states are extreme (alexisomia) |
| Multidimensional Assessment of Interoceptive Awareness* | MAIA | An 8-subscale state-trait self-report questionnaire to measure multiple dimensions of interoception (awareness of bodily sensations) |
| Noticing |  | Awareness of uncomfortable, comfortable, and neutral body sensations |
| Not-Distracting | ND | Tendency not to ignore or distract oneself from sensations of pain or discomfort |
| Not-Worrying | NW | Tendency not to worry or experience emotional distress with sensations of pain or discomfort |
| Attention Regulation | AR | Ability to sustain and control attention to body sensations |
| Emotional Awareness | EA | Awareness of the connection between body sensations and emotional states |
| Self-Regulation | SR | Ability to regulate distress by attention to body sensations |
| Body Listening | BL | Active listening to the body for insight |
| Trusting |  | Experience of one’s body as safe and trustworthy |
| Self-Awareness Questionnaire | SAQ | A self-report tool assessing interoceptive awareness |
| Factor 1 | F1 | Awareness of visceral sensations (e.g., heartbeat) |
| Factor 2 | F2 | Awareness of somatosensory sensations (e.g., pins and needles) |
| Total |  | Global interoceptive awareness; higher scores indicate higher awareness |
| Sensory Profile Interoception | SPI | A participation-focused measure of internal body sensations, evaluating how interoception manifests itself in everyday life behaviours |
| Avoiding |  | Active behaviours to avoid interoceptive sensations |
| Registration |  | Lack of awareness of interoceptive input |
| Seeking |  | Active behaviours to increase interoceptive input |
| Sensitivity |  | Heightened awareness of interoception |
| Three-Domain Interoceptive Sensations Questionnaire | THISQ | A three-scale questionnaire that assesses the perception of neutral sensations in respiratory, cardiac, and gastroesophageal domains |
| Cardio-Respiratory Activation | CRA | Self-perception of neutral cardiac and respiratory activation (e.g., faster heartrate) |
| Cardio-Respiratory Deactivation | CRD | Self-perception of neutral cardiac and respiratory deactivation (e.g., shallower breathing) |
| Gastro-esophageal Sensations | GES | Self-perception of neutral gastroesophageal sensations (e.g., bowel movements) |
| Total |  | Global perception of neutral respiratory, cardiac, and gastroesophageal sensations |
| * Abbreviation for Multidimensional Assessment of Interoceptive Awareness, Version 2: MAIA-2 | | |
