# Supplementary material for: A systematic review and meta-analysis of the relationship between subjective interoception and alexithymia: Implications for construct definitions and measurement
Source: PLoS One. 2024 Nov 7;19(11):e0310411. doi: 10.1371/journal.pone.0310411 (PMC11542822; doi:10.1371/journal.pone.0310411)
Supplement: S6 File — (DOCX) [file pone.0310411.s006.docx]

**Table S5. Sample characteristics and extracted correlations of each Independent Sample within included studies employing interoceptive self-report scales to examine their relationship with global alexithymia.**

| **Study** | **Interoception Scale** | **Alexithymia Scale** | ***N***  **(adj.)*** | ***Extracted***  ***r*** | **Clinical Status (0 = Non-Clinical,**  **1 = Clinical )** | **% Female** | **Sample Region** |
| --- | --- | --- | --- | --- | --- | --- | --- |
| Ricciardi (2021) - FMD/HC | BAQ | TAS-20-Total | 55 | -0.045 | 1 | 60 | Europe - UK |
| Zamariola (2018) - Study 2 | BAQ | TAS-20-Total | 158 | -0.19 | 0 | 74.7 | Europe - Other |
| Zamariola (2018) - Study 3 | BAQ | TAS-20-Total | 157 | -0.15 | 0 | 75.2 | Europe - Other |
| Betka (2018) | BPQ-BA | TAS-20-Total | 590 | 0.20 | 0 | 74 | Europe - UK |
| Brand (2022) | BPQ-BA | TAS-20-Total | 614 | -0.077 | 0 | 66 | Europe - Other |
| Campos (2021) | BPQ-BA | TAS-20-Total | 515 | -0.03 | 0 | 60 | Europe - Other |
| Ernst (2014) | BPQ-BA | TAS-20-Total | 18 | 0.55 | 0 | 59 | Europe - Other |
| Gaggero (2021) - ITa | BPQ-BA | TAS-20-Total | 162.5* | -0.09 | 0 | 68 | Europe - Other |
| Gaggero (2021) - ITb | BPQ-BA | BVAQ-C | 162.5* | -0.17 | 0 | 68 | Europe - Other |
| Gaggero (2021) - USa | BPQ-BA | TAS-20-Total | 125* | 0.07 | 0 | 68 | North America |
| Gaggero (2021) - USb | BPQ-BA | BVAQ-C | 125* | 0.02 | 0 | 68 | North America |
| Gaggero (2021) - SGa | BPQ-BA | TAS-20-Total | 119.5* | -0.02 | 0 | 62.8 | Asia |
| Gaggero (2021) - SGb | BPQ-BA | BVAQ-C | 119.5* | -0.09 | 0 | 62.8 | Asia |
| Hassen (2023) - ASD | BPQ-BA | TAS-20-Total | 27 | -0.51 | 1 | 50 | Europe - Other |
| Hassen (2023) - Sample 1 | BPQ-BA | TAS-20-Total | 30 | -0.05 | 0 | 46.7 | Europe - Other |
| Hassen (2023) - Sample 2 | BPQ-BA | TAS-20-Total | 20 | -0.112 | 0 | 80 | Europe - Other |
| Murphy (2020) - Study 2 | BPQ-BA | TAS-20-Total | 76 | 0.08 | 0 | 61.7 | Europe - UK |
| Murphy (2020) - Study 5 | BPQ-BA | TAS-20-Total | 35 | 0.07 | 0 | 74.1 | Europe - UK |
| Brand (2022) | BPQ-R-Sub | TAS-20-Total | 614 | 0.166 | 0 | 66 | Europe - Other |
| Gaggero (2021) - ITa | BPQ-R-Sub | TAS-20-Total | 162.5* | 0.23 | 0 | 68 | Europe - Other |
| Gaggero (2021) - ITb | BPQ-R-Sub | BVAQ-C | 162.5* | 0.19 | 0 | 68 | Europe - Other |
| Gaggero (2021) - USa | BPQ-R-Sub | TAS-20-Total | 125* | 0.32 | 0 | 68 | North America |
| Gaggero (2021) - USb | BPQ-R-Sub | BVAQ-C | 125* | 0.2 | 0 | 68 | North America |
| Gaggero (2021) - SGa | BPQ-R-Sub | TAS-20-Total | 119.5* | 0.28 | 0 | 62.8 | Asia |
| Gaggero (2021) - SGb | BPQ-R-Sub | BVAQ-C | 119.5* | 0.19 | 0 | 62.8 | Asia |
| Brand (2022) | BPQ-R-Supra | TAS-20-Total | 614 | 0.309 | 0 | 66 | Europe - Other |
| Gaggero (2021) - ITa | BPQ-R-Supra | TAS-20-Total | 162.5* | 0.25 | 0 | 68 | Europe - Other |
| Gaggero (2021) - ITb | BPQ-R-Supra | BVAQ-C | 162.5* | 0.25 | 0 | 68 | Europe - Other |
| Gaggero (2021) - USa | BPQ-R-Supra | TAS-20-Total | 125* | 0.48 | 0 | 68 | North America |
| Gaggero (2021) - USb | BPQ-R-Supra | BVAQ-C | 125* | 0.4 | 0 | 68 | North America |
| Gaggero (2021) - SGa | BPQ-R-Supra | TAS-20-Total | 119.5* | 0.37 | 0 | 62.8 | Asia |
| Gaggero (2021) - SGb | BPQ-R-Supra | BVAQ-C | 119.5* | 0.33 | 0 | 62.8 | Asia |
| Campos (2021) | BPQ-R-Total | TAS-20-Total | 515 | 0.37 | 0 | 60 | Europe - Other |
| Ernst (2014) | BPQ-R-Total | TAS-20-Total | 18 | 0.65 | 0 | 59 | Europe - Other |
| Gaggero (2021) - ITa | BPQ-R-Total | TAS-20-Total | 162.5* | 0.28 | 0 | 68 | Europe - Other |
| Gaggero (2021) - ITb | BPQ-R-Total | BVAQ-C | 162.5* | 0.26 | 0 | 68 | Europe - Other |
| Gaggero (2021) - USa | BPQ-R-Total | TAS-20-Total | 125* | 0.46 | 0 | 68 | North America |
| Gaggero (2021) - USb | BPQ-R-Total | BVAQ-C | 125* | 0.37 | 0 | 68 | North America |
| Gaggero (2021) - SGa | BPQ-R-Total | TAS-20-Total | 119.5* | 0.38 | 0 | 62.8 | Asia |
| Gaggero (2021) - SGb | BPQ-R-Total | BVAQ-C | 119.5* | 0.33 | 0 | 62.8 | Asia |
| Taylor (1996) - AN | EDI-IAw | TAS-20-Total | 48 | 0.42 | 1 | 100 | Europe - UK |
| Taylor (1996) - Sample 1 | EDI-IAw | TAS-20-Total | 30 | 0.16 | 0 | 100 | Europe - UK |
| Taylor (1996) - Sample 2 | EDI-IAw | TAS-20-Total | 116 | 0.33 | 0 | 100 | Europe - UK |
| Taylor (1996) - Sample 3 | EDI-IAw | TAS-20-Total | 118 | 0.13 | 0 | 0 | Europe - UK |
| Brand (2022) | IAS | TAS-20-Total | 614 | -0.29 | 0 | 66 | Europe - Other |
| Campos (2021) | IAS | TAS-20-Total | 515 | -0.29 | 0 | 60 | Europe - Other |
| Gaggero (2021) - ITa | IAS | TAS-20-Total | 162.5* | -0.31 | 0 | 68 | Europe - Other |
| Gaggero (2021) - ITb | IAS | BVAQ-C | 162.5* | -0.34 | 0 | 68 | Europe - Other |
| Gaggero (2021) - USa | IAS | TAS-20-Total | 125* | -0.34 | 0 | 68 | North America |
| Gaggero (2021) - USb | IAS | BVAQ-C | 125* | -0.32 | 0 | 68 | North America |
| Gaggero (2021) - SGa | IAS | TAS-20-Total | 119.5* | -0.22 | 0 | 62.8 | Asia |
| Gaggero (2021) - SGb | IAS | BVAQ-C | 119.5* | -0.24 | 0 | 62.8 | Asia |
| Jakobson (2021) | IAS | TAS-20-Total | 209 | -0.27 | 0 | 55.7 | North America |
| Murphy (2020) - Study 2 | IAS | TAS-20-Total | 76 | -0.255 | 0 | 61.7 | Europe - UK |
| Murphy (2020) - Study 5 | IAS | TAS-20-Total | 35 | -0.572 | 0 | 74.1 | Europe - UK |
| Tünte (2022) - Sample 2 | IATS-Total | TAS-20-Total | 447 | 0.22 | 0 | Unclear | Europe - Other |
| Tünte (2022) - Sample 1 | IATS-Total | TAS-20-Total | 134 | 0.21 | 0 | Unclear | Europe - Other |
| Brand (2022) | ICQ-Total | TAS-20-Total | 614 | 0.52 | 0 | 66 | Europe - Other |
| Brewer (2016) | ICQ-Total | TAS-20-Total | 653 | 0.69 | 0 | 51 | Europe - UK |
| Gaggero (2021) - ITa | ICQ-Total | TAS-20-Total | 162.5* | 0.46 | 0 | 68 | Europe - Other |
| Gaggero (2021) - ITb | ICQ-Total | BVAQ-C | 162.5* | 0.5 | 0 | 68 | Europe - Other |
| Gaggero (2021) - USa | ICQ-Total | TAS-20-Total | 125* | 0.69 | 0 | 68 | North America |
| Gaggero (2021) - USb | ICQ-Total | BVAQ-C | 125* | 0.62 | 0 | 68 | North America |
| Gaggero (2021) - SGa | ICQ-Total | TAS-20-Total | 119.5* | 0.46 | 0 | 62.8 | Asia |
| Gaggero (2021) - SGb | ICQ-Total | BVAQ-C | 119.5* | 0.46 | 0 | 62.8 | Asia |
| Murphy (2020) - Study 5 | ICQ-Total | TAS-20-Total | 35 | 0.648 | 0 | 74.1 | Europe - UK |
| Bonete (2023) - ASD | ISQ-Total | TAS-20-Total | 33 | 0.502 | 0 | 0 | Europe - Other |
| Bonete (2023) | ISQ-Total | TAS-20-Total | 35 | 0.563 | 0 | 0 | Europe - Other |
| Fiene (2018) | ISQ-Total | TAS-20-Total | 511 | 0.76 | 0 | 61.3 | Australasia |
| Brand (2022) | MAIA-AR | TAS-20-Total | 614 | -0.31 | 0 | 66 | Europe - Other |
| Da Costa Silva (2022) | MAIA-AR | TAS-20-Total | 308 | -0.37 | 0 | 61.4 | Europe - Other |
| Desdentado (2022) | MAIA-AR | TAS-20-Total | 391 | -0.26 | 0 | 61.4 | Europe - Other |
| Edwards & Lowe (2021) | MAIA-AR | TAS-20-Total | 230 | -0.27 | 0 | 51 | Europe - UK |
| Gaggero (2021) - ITa | MAIA-AR | TAS-20-Total | 162.5* | -0.29 | 0 | 68 | Europe - Other |
| Gaggero (2021) - ITb | MAIA-AR | BVAQ-C | 162.5* | -0.34 | 0 | 68 | Europe - Other |
| Gaggero (2021) - USa | MAIA-AR | TAS-20-Total | 125* | -0.23 | 0 | 68 | North America |
| Gaggero (2021) - USb | MAIA-AR | BVAQ-C | 125* | -0.32 | 0 | 68 | North America |
| Gaggero (2021) - SGa | MAIA-AR | TAS-20-Total | 119.5* | -0.18 | 0 | 62.8 | Asia |
| Gaggero (2021) - SGb | MAIA-AR | BVAQ-C | 119.5* | -0.21 | 0 | 62.8 | Asia |
| Huang (2022) | MAIA-AR | TAS-20-Total | 224 | -0.18 | 0 | 70.1 | Asia |
| Lyvers & Thornberg (2023) | MAIA-AR | TAS-20-Total | 337 | -0.20 | 0 | 66 | Australasia |
| Mul (2018) | MAIA-AR | TAS-20-Total | 52 | -0.34 | 1 | 26.9 | Europe - UK |
| Pink (2021) | MAIA-AR | TAS-20-Total | 172 | -0.25 | 0 | 100 | Europe - UK |
| Schmitz (2021) - FM | MAIA-AR | TAS-20-Total | 55 | -0.34 | 1 | 83.9 | Europe - Other |
| Schmitz (2021) | MAIA-AR | TAS-20-Total | 55 | -0.16 | 0 | 83.9 | Europe - Other |
| Vinni (2023) - CD | MAIA-AR | TAS-20-Total | 41 | -0.32 | 1 | 36.8 | Europe - Other |
| Vinni (2023) - UC | MAIA-AR | TAS-20-Total | 16 | -0.16 | 1 | 62.5 | Europe - Other |
| Zahid (2023) | MAIA-AR | TAS-20-Total | 379.5* | -0.25 | 0 | 50.6 | North America |
| Zahid (2023) | MAIA-AR | PAQ-Total | 379.5* | -0.18 | 0 | 50.6 | North America |
| Zamariola (2018) - Studies 4-6 | MAIA-AR | TAS-20-Total | 263 | -0.33 | 0 | 77.9 | Europe - Other |
| Brand (2022) | MAIA-BL | TAS-20-Total | 614 | -0.18 | 0 | 66 | Europe - Other |
| Da Costa Silva (2022) | MAIA-BL | TAS-20-Total | 308 | -0.33 | 0 | 61.4 | Europe - Other |
| Desdentado (2022) | MAIA-BL | TAS-20-Total | 391 | -0.22 | 0 | 61.4 | Europe - Other |
| Edwards & Lowe (2021) | MAIA-BL | TAS-20-Total | 230 | -0.22 | 0 | 51 | Europe - UK |
| Gaggero (2021) - ITa | MAIA-BL | TAS-20-Total | 162.5* | -0.30 | 0 | 68 | Europe - Other |
| Gaggero (2021) - ITb | MAIA-BL | BVAQ-C | 162.5* | -0.39 | 0 | 68 | Europe - Other |
| Gaggero (2021) - USa | MAIA-BL | TAS-20-Total | 125* | -0.25 | 0 | 68 | North America |
| Gaggero (2021) - USb | MAIA-BL | BVAQ-C | 125* | -0.34 | 0 | 68 | North America |
| Gaggero (2021) - SGa | MAIA-BL | TAS-20-Total | 119.5* | -0.1 | 0 | 62.8 | Asia |
| Gaggero (2021) - SGb | MAIA-BL | BVAQ-C | 119.5* | -0.2 | 0 | 62.8 | Asia |
| Huang (2022) | MAIA-BL | TAS-20-Total | 224 | -0.14 | 0 | 70.1 | Asia |
| Pink (2021) | MAIA-BL | TAS-20-Total | 172 | -0.08 | 0 | 100 | Europe - UK |
| Schmitz (2021) - FM | MAIA-BL | TAS-20-Total | 55 | -0.29 | 1 | 83.9 | Europe - Other |
| Schmitz (2021) | MAIA-BL | TAS-20-Total | 55 | -0.28 | 0 | 83.9 | Europe - Other |
| Vinni (2023) - CD | MAIA-BL | TAS-20-Total | 41 | -0.35 | 1 | 36.8 | Europe - Other |
| Vinni (2023) - UC | MAIA-BL | TAS-20-Total | 16 | -0.02 | 1 | 62.5 | Europe - Other |
| Zahid (2023) | MAIA-BL | TAS-20-Total | 379.5* | -.21 | 0 | 50.6 | North America |
| Zahid (2023) | MAIA-BL | PAQ-Total | 379.5* | -.21 | 0 | 50.6 | North America |
| Zamariola (2018) - Studies 4-6 | MAIA-BL | TAS-20-Total | 263 | -0.23 | 0 | 77.9 | Europe - Other |
| Brand (2022) | MAIA-EA | TAS-20-Total | 614 | -0.21 | 0 | 66 | Europe - Other |
| Da Costa Silva (2022) | MAIA-EA | TAS-20-Total | 308 | -0.30 | 0 | 61.4 | Europe - Other |
| Desdentado (2022) | MAIA-EA | TAS-20-Total | 391 | -0.15 | 0 | 61.4 | Europe - Other |
| Edwards & Lowe (2021) | MAIA-EA | TAS-20-Total | 230 | -0.17 | 0 | 51 | Europe - UK |
| Gaggero (2021) - ITa | MAIA-EA | TAS-20-Total | 162.5* | -0.18 | 0 | 68 | Europe - Other |
| Gaggero (2021) - ITb | MAIA-EA | BVAQ-C | 162.5* | -0.24 | 0 | 68 | Europe - Other |
| Gaggero (2021) - USa | MAIA-EA | TAS-20-Total | 125* | -0.2 | 0 | 68 | North America |
| Gaggero (2021) - USb | MAIA-EA | BVAQ-C | 125* | -0.27 | 0 | 68 | North America |
| Gaggero (2021) - SGa | MAIA-EA | TAS-20-Total | 119.5* | -0.1 | 0 | 62.8 | Asia |
| Gaggero (2021) - SGb | MAIA-EA | BVAQ-C | 119.5* | -0.17 | 0 | 62.8 | Asia |
| Huang (2022) | MAIA-EA | TAS-20-Total | 224 | -0.13 | 0 | 70.1 | Asia |
| Pink (2021) | MAIA-EA | TAS-20-Total | 172 | -0.18 | 0 | 100 | Europe - UK |
| Schmitz (2021) - FM | MAIA-EA | TAS-20-Total | 55 | -0.10 | 1 | 83.9 | Europe - Other |
| Schmitz (2021) | MAIA-EA | TAS-20-Total | 55 | -0.33 | 0 | 83.9 | Europe - Other |
| Vinni (2023) - CD | MAIA-EA | TAS-20-Total | 41 | -0.17 | 1 | 36.8 | Europe - Other |
| Vinni (2023) - UC | MAIA-EA | TAS-20-Total | 16 | -0.01 | 1 | 62.5 | Europe - Other |
| Zamariola (2018) - Studies 4-6 | MAIA-EA | TAS-20-Total | 263 | -0.07 | 0 | 77.9 | Europe - Other |
| Brand (2022) | MAIA-ND | TAS-20-Total | 614 | -0.33 | 0 | 66 | Europe - Other |
| Da Costa Silva (2022) | MAIA-ND | TAS-20-Total | 308 | -0.27 | 0 | 61.4 | Europe - Other |
| Desdentado (2022) | MAIA-ND | TAS-20-Total | 391 | -0.16 | 0 | 61.4 | Europe - Other |
| Edwards & Lowe (2021) | MAIA-ND | TAS-20-Total | 230 | -0.30 | 0 | 51 | Europe - UK |
| Gaggero (2021) - ITa | MAIA-ND | TAS-20-Total | 162.5* | -0.15 | 0 | 68 | Europe - Other |
| Gaggero (2021) - ITb | MAIA-ND | BVAQ-C | 162.5* | -0.21 | 0 | 68 | Europe - Other |
| Gaggero (2021) - USa | MAIA-ND | TAS-20-Total | 125* | -0.31 | 0 | 68 | North America |
| Gaggero (2021) - USb | MAIA-ND | BVAQ-C | 125* | -0.25 | 0 | 68 | North America |
| Gaggero (2021) - SGa | MAIA-ND | TAS-20-Total | 119.5* | -0.30 | 0 | 62.8 | Asia |
| Gaggero (2021) - SGb | MAIA-ND | BVAQ-C | 119.5* | -0.25 | 0 | 62.8 | Asia |
| Huang (2022) | MAIA-ND | TAS-20-Total | 224 | -0.14 | 0 | 70.1 | Asia |
| Lyvers & Thornberg (2023) | MAIA-ND | TAS-20-Total | 337 | -0.15 | 0 | 66 | Australasia |
| Pink (2021) | MAIA-ND | TAS-20-Total | 172 | -0.07 | 0 | 100 | Europe - UK |
| Schmitz (2021) - FM | MAIA-ND | TAS-20-Total | 55 | -0.20 | 1 | 83.9 | Europe - Other |
| Schmitz (2021) | MAIA-ND | TAS-20-Total | 55 | -0.18 | 0 | 83.9 | Europe - Other |
| Vinni (2023) - CD | MAIA-ND | TAS-20-Total | 41 | 0.1 | 1 | 36.8 | Europe - Other |
| Vinni (2023) - UC | MAIA-ND | TAS-20-Total | 16 | -0.16 | 1 | 62.5 | Europe - Other |
| Zamariola (2018) - Studies 4-6 | MAIA-ND | TAS-20-Total | 263 | -0.11 | 0 | 77.9 | Europe - Other |
| Brand (2022) | MAIA-Noticing | TAS-20-Total | 614 | -0.13 | 0 | 66 | Europe - Other |
| Da Costa Silva (2022) | MAIA-Noticing | TAS-20-Total | 308 | -0.30 | 0 | 61.4 | Europe - Other |
| Desdentado (2022) | MAIA-Noticing | TAS-20-Total | 391 | -0.08 | 0 | 61.4 | Europe - Other |
| Edwards & Lowe (2021) | MAIA-Noticing | TAS-20-Total | 230 | -0.18 | 0 | 51 | Europe - UK |
| Gaggero (2021) - ITa | MAIA-Noticing | TAS-20-Total | 162.5* | -0.22 | 0 | 68 | Europe - Other |
| Gaggero (2021) - ITb | MAIA-Noticing | BVAQ-C | 162.5* | -0.26 | 0 | 68 | Europe - Other |
| Gaggero (2021) - USa | MAIA-Noticing | TAS-20-Total | 125* | -0.24 | 0 | 68 | North America |
| Gaggero (2021) - USb | MAIA-Noticing | BVAQ-C | 125* | -0.32 | 0 | 68 | North America |
| Gaggero (2021) - SGa | MAIA-Noticing | TAS-20-Total | 119.5* | -0.03 | 0 | 62.8 | Asia |
| Gaggero (2021) - SGb | MAIA-Noticing | BVAQ-C | 119.5* | -0.13 | 0 | 62.8 | Asia |
| Huang (2022) | MAIA-Noticing | TAS-20-Total | 224 | -0.12 | 0 | 70.1 | Asia |
| Pink (2021) | MAIA-Noticing | TAS-20-Total | 172 | -0.13 | 0 | 100 | Europe - UK |
| Schmitz (2021) - FM | MAIA-Noticing | TAS-20-Total | 55 | -0.27 | 1 | 83.9 | Europe - Other |
| Schmitz (2021) | MAIA-Noticing | TAS-20-Total | 55 | -.18 | 0 | 83.9 | Europe - Other |
| Vinni (2023) - CD | MAIA-Noticing | TAS-20-Total | 41 | 0.01 | 1 | 36.8 | Europe - Other |
| Vinni (2023) - UC | MAIA-Noticing | TAS-20-Total | 16 | -0.32 | 1 | 62.5 | Europe - Other |
| Zamariola (2018) - Studies 4-6 | MAIA-Noticing | TAS-20-Total | 263 | -0.23 | 0 | 77.9 | Europe - Other |
| Brand (2022) | MAIA-NW | TAS-20-Total | 614 | -0.22 | 0 | 66 | Europe - Other |
| Da Costa Silva (2022) | MAIA-NW | TAS-20-Total | 308 | 0.02 | 0 | 61.4 | Europe - Other |
| Desdentado (2022) | MAIA-NW | TAS-20-Total | 391 | -0.18 | 0 | 61.4 | Europe - Other |
| Edwards & Lowe (2021) | MAIA-NW | TAS-20-Total | 230 | -0.02 | 0 | 51 | Europe - UK |
| Gaggero (2021) - ITa | MAIA-NW | TAS-20-Total | 162.5* | -0.18 | 0 | 68 | Europe - Other |
| Gaggero (2021) - ITb | MAIA-NW | BVAQ-C | 162.5* | -0.07 | 0 | 68 | Europe - Other |
| Gaggero (2021) - USa | MAIA-NW | TAS-20-Total | 125* | -0.23 | 0 | 68 | North America |
| Gaggero (2021) - USb | MAIA-NW | BVAQ-C | 125* | -0.1 | 0 | 68 | North America |
| Gaggero (2021) - SGa | MAIA-NW | TAS-20-Total | 119.5* | -0.25 | 0 | 62.8 | Asia |
| Gaggero (2021) - SGb | MAIA-NW | BVAQ-C | 119.5* | -0.09 | 0 | 62.8 | Asia |
| Huang (2022) | MAIA-NW | TAS-20-Total | 224 | -0.14 | 0 | 70.1 | Asia |
| Pink (2021) | MAIA-NW | TAS-20-Total | 172 | -0.17 | 0 | 100 | Europe - UK |
| Schmitz (2021) - FM | MAIA-NW | TAS-20-Total | 55 | -0.42 | 1 | 83.9 | Europe - Other |
| Schmitz (2021) | MAIA-NW | TAS-20-Total | 55 | -0.29 | 0 | 83.9 | Europe - Other |
| Vinni (2023) - CD | MAIA-NW | TAS-20-Total | 41 | -0.34 | 1 | 36.8 | Europe - Other |
| Vinni (2023) - UC | MAIA-NW | TAS-20-Total | 16 | 0.04 | 1 | 62.5 | Europe - Other |
| Zamariola (2018) - Studies 4-6 | MAIA-NW | TAS-20-Total | 263 | -0.33 | 0 | 77.9 | Europe - Other |
| Brand (2022) | MAIA-SR | TAS-20-Total | 614 | -0.34 | 0 | 66 | Europe - Other |
| Da Costa Silva (2022) | MAIA-SR | TAS-20-Total | 308 | -0.37 | 0 | 61.4 | Europe - Other |
| Desdentado (2022) | MAIA-SR | TAS-20-Total | 391 | -0.16 | 0 | 61.4 | Europe - Other |
| Edwards & Lowe (2021) | MAIA-SR | TAS-20-Total | 230 | -0.17 | 0 | 51 | Europe - UK |
| Gaggero (2021) - ITa | MAIA-SR | TAS-20-Total | 162.5* | -0.28 | 0 | 68 | Europe - Other |
| Gaggero (2021) - ITb | MAIA-SR | BVAQ-C | 162.5* | -0.26 | 0 | 68 | Europe - Other |
| Gaggero (2021) - USa | MAIA-SR | TAS-20-Total | 125* | -0.34 | 0 | 68 | North America |
| Gaggero (2021) - USb | MAIA-SR | BVAQ-C | 125* | -0.39 | 0 | 68 | North America |
| Gaggero (2021) - SGa | MAIA-SR | TAS-20-Total | 119.5* | -0.25 | 0 | 62.8 | Asia |
| Gaggero (2021) - SGb | MAIA-SR | BVAQ-C | 119.5* | -0.32 | 0 | 62.8 | Asia |
| Huang (2022) | MAIA-SR | TAS-20-Total | 224 | -0.22 | 0 | 70.1 | Asia |
| Pink (2021) | MAIA-SR | TAS-20-Total | 172 | -0.21 | 0 | 100 | Europe - UK |
| Schmitz (2021) - FM | MAIA-SR | TAS-20-Total | 55 | -0.38 | 1 | 83.9 | Europe - Other |
| Schmitz (2021) | MAIA-SR | TAS-20-Total | 55 | -0.30 | 0 | 83.9 | Europe - Other |
| Vinni (2023) - CD | MAIA-SR | TAS-20-Total | 41 | -0.34 | 1 | 36.8 | Europe - Other |
| Vinni (2023) - UC | MAIA-SR | TAS-20-Total | 16 | -0.03 | 1 | 62.5 | Europe - Other |
| Zahid (2023) | MAIA-SR | TAS-20-Total | 379.5* | -0.29 | 0 | 50.6 | North America |
| Zahid (2023) | MAIA-SR | PAQ-Total | 379.5* | -0.19 | 0 | 50.6 | North America |
| Zamariola (2018) - Studies 4-6 | MAIA-SR | TAS-20-Total | 263 | -0.28 | 0 | 77.9 | Europe - Other |
| Berenguer (2023) - F | MAIA-Total | TAS-20-Total | 152 | -0.36 | 0 | 100 | Europe - Other |
| Berenguer (2023) - M | MAIA-Total | TAS-20-Total | 86 | -0.3 | 0 | 0 | Europe - Other |
| Da Costa Silva (2022) | MAIA-Total | TAS-20-Total | 308 | -0.50 | 0 | 61.4 | Europe - Other |
| Ferraro & Taylor (2021) | MAIA-Total | TAS-20-Total | 219 | 0.31 | 0 | 22 | Australasia |
| Gaggero (2021) - ITa | MAIA-Total | TAS-20-Total | 162.5* | -0.4 | 0 | 68 | Europe - Other |
| Gaggero (2021) - ITb | MAIA-Total | BVAQ-C | 162.5* | -0.44 | 0 | 68 | Europe - Other |
| Gaggero (2021) - USa | MAIA-Total | TAS-20-Total | 125* | -0.42 | 0 | 68 | North America |
| Gaggero (2021) - USb | MAIA-Total | BVAQ-C | 125* | -0.46 | 0 | 68 | North America |
| Gaggero (2021) - SGa | MAIA-Total | TAS-20-Total | 119.5* | -0.36 | 0 | 62.8 | Asia |
| Gaggero (2021) - SGb | MAIA-Total | BVAQ-C | 119.5* | -0.43 | 0 | 62.8 | Asia |
| Morales (2022) | MAIA-Total | TAS-20-Total | 128 | -0.26 | 0 | 100 | North America |
| Sweetnam & Flack (2023) | MAIA-Total | TAS-20-Total | 404 | -0.55 | 0 | 86.4 | Australasia |
| Brand (2022) | MAIA-Trusting | TAS-20-Total | 614 | -0.4 | 0 | 66 | Europe - Other |
| Da Costa Silva (2022) | MAIA-Trusting | TAS-20-Total | 308 | -0.42 | 0 | 61.4 | Europe - Other |
| Desdentado (2022) | MAIA-Trusting | TAS-20-Total | 391 | -0.28 | 0 | 61.4 | Europe - Other |
| Edwards & Lowe (2021) | MAIA-Trusting | TAS-20-Total | 230 | -0.26 | 0 | 51 | Europe - UK |
| Gaggero (2021) - ITa | MAIA-Trusting | TAS-20-Total | 162.5* | -0.35 | 0 | 68 | Europe - Other |
| Gaggero (2021) - ITb | MAIA-Trusting | BVAQ-C | 162.5* | -0.39 | 0 | 68 | Europe - Other |
| Gaggero (2021) - USa | MAIA-Trusting | TAS-20-Total | 125* | -0.39 | 0 | 68 | North America |
| Gaggero (2021) - USb | MAIA-Trusting | BVAQ-C | 125* | -0.42 | 0 | 68 | North America |
| Gaggero (2021) - SGa | MAIA-Trusting | TAS-20-Total | 119.5* | -0.36 | 0 | 62.8 | Asia |
| Gaggero (2021) - SGb | MAIA-Trusting | BVAQ-C | 119.5* | -0.4 | 0 | 62.8 | Asia |
| Huang (2022) | MAIA-Trusting | TAS-20-Total | 224 | -0.27 | 0 | 70.1 | Asia |
| Lyvers & Thornberg (2023) | MAIA-Trusting | TAS-20-Total | 337 | -0.27 | 0 | 66 | Australasia |
| Pink (2021) | MAIA-Trusting | TAS-20-Total | 172 | -0.33 | 0 | 100 | Europe - UK |
| Schmitz (2021) - FM | MAIA-Trusting | TAS-20-Total | 55 | -0.23 | 1 | 83.9 | Europe - Other |
| Schmitz (2021) | MAIA-Trusting | TAS-20-Total | 55 | -0.30 | 0 | 83.9 | Europe - Other |
| Vinni (2023) - CD | MAIA-Trusting | TAS-20-Total | 41 | -0.31 | 1 | 36.8 | Europe - Other |
| Vinni (2023) - UC | MAIA-Trusting | TAS-20-Total | 16 | -0.1 | 1 | 62.5 | Europe - Other |
| Zamariola (2018) - Studies 4-6 | MAIA-Trusting | TAS-20-Total | 263 | -0.39 | 0 | 77.9 | Europe - Other |
