# Supplementary material for: A systematic review and meta-analysis of the relationship between subjective interoception and alexithymia: Implications for construct definitions and measurement
Source: PLoS One. 2024 Nov 7;19(11):e0310411. doi: 10.1371/journal.pone.0310411 (PMC11542822; doi:10.1371/journal.pone.0310411)
Supplement: S7 File — (DOCX) [file pone.0310411.s007.docx]

**Table S6. Sample characteristics and extracted correlations of each Independent Sample within included studies employing interoceptive self-report scales to examine their relationship with DIF.**

| **Study** | **Interoception Scale** | **n** | ***Extracted r*** | **Clinical Status (0 = Non-Clinical,**  **1 = Clinical )** | **% Female** | **Sample Region** |
| --- | --- | --- | --- | --- | --- | --- |
| Zamariola (2018) - Study 2 | BAQ-Total | 158 | -0.08 | 0 | 74.7 | Europe - Other |
| Zamariola (2018) - Study 3 | BAQ-Total | 157 | -0.06 | 0 | 75.2 | Europe - Other |
| Betka (2018) | BPQ-BA | 590 | 0.23 | 0 | 74 | Europe - UK |
| Brand (2022) | BPQ-BA | 614 | -0.005 | 0 | 66 | Europe - Other |
| Gaggero (2021) - IT | BPQ-BA | 325 | -0.03 | 0 | 68 | Europe - Other |
| Gaggero (2021) - US | BPQ-BA | 250 | 0.1 | 0 | 68 | North America |
| Gaggero (2021) - SG | BPQ-BA | 239 | -0.03 | 0 | 62.8 | Asia |
| Hassen (2023) - ASD | BPQ-BA | 27 | -0.482 | 0 | 50 | Europe - Other |
| Hassen (2023) - Sample 1 | BPQ-BA | 30 | -0.01 | 0 | 70.1 | Europe - Other |
| Hassen (2023) - Sample 2 | BPQ-BA | 20 | -0.15 | 0 | 80 | Europe - Other |
| Brand (2022) | BPQ-R-Sub | 614 | 0.111 | 0 | 66 | Europe - Other |
| Gaggero (2021) - IT | BPQ-R-Sub | 325 | 0.37 | 0 | 68 | Europe - Other |
| Gaggero (2021) - US | BPQ-R-Sub | 250 | 0.4 | 0 | 68 | North America |
| Gaggero (2021) - SG | BPQ-R-Sub | 239 | 0.34 | 0 | 62.8 | Asia |
| Brand (2022) | BPQ-R-Supra | 614 | 0.23 | 0 | 66 | Europe - Other |
| Gaggero (2021) - IT | BPQ-R-Supra | 325 | 0.4 | 0 | 68 | Europe - Other |
| Gaggero (2021) - US | BPQ-R-Supra | 250 | 0.55 | 0 | 68 | North America |
| Gaggero (2021) - SG | BPQ-R-Supra | 239 | 0.4 | 0 | 62.8 | Asia |
| Gaggero (2021) - IT | BPQ-R-Total | 325 | 0.45 | 0 | 68 | Europe - Other |
| Gaggero (2021) - US | BPQ-R-Total | 250 | 0.55 | 0 | 68 | North America |
| Gaggero (2021) - SG | BPQ-R-Total | 239 | 0.43 | 0 | 62.8 | Asia |
| Brand (2022) | IAS-Total | 614 | -0.3 | 0 | 66 | Europe - Other |
| Gaggero (2021) - IT | IAS-Total | 323 | -0.33 | 0 | 68 | Europe - Other |
| Gaggero (2021) - US | IAS-Total | 248 | -0.33 | 0 | 68 | North America |
| Gaggero (2021) - SG | IAS-Total | 239 | -0.28 | 0 | 62.8 | Asia |
| Jakobson & Rigby (2021) | IAS-Total | 209 | -0.2 | 0 |  | North America |
| Tünte (2022) - Sample 2 | IATS-Total | 447 | 0.28 | 0 |  | Europe - Other |
| Tünte (2022) - Sample 1 | IATS-Total | 134 | 0.24 | 0 |  | Europe - Other |
| Brand (2022) | ICQ-Total | 614 | 0.52 | 0 | 66 | Europe - Other |
| Gaggero (2021) - IT | ICQ-Total | 325 | 0.5 | 0 | 68 | Europe - Other |
| Gaggero (2021) - US | ICQ-Total | 250 | 0.71 | 0 | 68 | North America |
| Gaggero (2021) - SG | ICQ-Total | 239 | 0.5 | 0 | 62.8 | Asia |
| Brand (2022) | MAIA-AR | 614 | -0.36 | 0 | 66 | Europe - Other |
| Da Costa Silva (2022) | MAIA-AR | 308 | -0.3 | 0 | 61.4 | Europe - Other |
| Desdentado (2022) | MAIA-AR | 391 | -0.20 | 0 | 61.4 | Europe - Other |
| Edwards & Lowe (2021) | MAIA-AR | 230 | -0.20 | 0 | 51 | Europe - UK |
| Gaggero (2021) - IT | MAIA-AR | 325 | -0.28 | 0 | 68 | Europe - Other |
| Gaggero (2021) - US | MAIA-AR | 250 | -0.14 | 0 | 68 | North America |
| Gaggero (2021) - SG | MAIA-AR | 239 | -0.11 | 0 | 62.8 | Asia |
| Pink (2021) | MAIA-AR | 172 | -0.82 | 0 | 100 | Europe - UK |
| Vinni (2023) - CD | MAIA-AR | 41 | -0.4 | 1 | 36.8 | Europe - Other |
| Vinni (2023) - UC | MAIA-AR | 16 | 0.11 | 1 | 62.5 | Europe - Other |
| Zamariola (2018) - Studies 4-6 | MAIA-AR | 263 | -0.32 | 0 | 77.9 | Europe - Other |
| Brand (2022) | MAIA-BL | 614 | -0.12 | 0 | 66 | Europe - Other |
| Da Costa Silva (2022) | MAIA-BL | 308 | -0.12 | 0 | 61.4 | Europe - Other |
| Desdentado (2022) | MAIA-BL | 391 | -0.12 | 0 | 61.4 | Europe - Other |
| Edwards & Lowe (2021) | MAIA-BL | 230 | -0.15 | 0 | 51 | Europe - UK |
| Gaggero (2021) - IT | MAIA-BL | 325 | -0.21 | 0 | 68 | Europe - Other |
| Gaggero (2021) - US | MAIA-BL | 250 | -0.13 | 0 | 68 | North America |
| Gaggero (2021) - SG | MAIA-BL | 239 | -0.06 | 0 | 62.8 | Asia |
| Pink (2021) | MAIA-BL | 172 | -0.05 | 0 | 100 | Europe - UK |
| Vinni (2023) - CD | MAIA-BL | 41 | -0.4 | 1 | 36.8 | Europe - Other |
| Vinni (2023) - UC | MAIA-BL | 16 | 0.43 | 1 | 62.5 | Europe - Other |
| Zamariola (2018) - Studies 4-6 | MAIA-BL | 263 | -0.11 | 0 | 77.9 | Europe - Other |
| Brand (2022) | MAIA-EA | 614 | -0.1 | 0 | 66 | Europe - Other |
| Da Costa Silva (2022) | MAIA-EA | 308 | -0.12 | 0 | 61.4 | Europe - Other |
| Desdentado (2022) | MAIA-EA | 391 | 0.01 | 0 | 61.4 | Europe - Other |
| Edwards & Lowe (2021) | MAIA-EA | 230 | -0.20 | 0 | 51 | Europe - UK |
| Gaggero (2021) - IT | MAIA-EA | 325 | -0.07 | 0 | 68 | Europe - Other |
| Gaggero (2021) - US | MAIA-EA | 250 | -0.12 | 0 | 68 | North America |
| Gaggero (2021) - SG | MAIA-EA | 239 | -0.05 | 0 | 62.8 | Asia |
| Pink (2021) | MAIA-EA | 172 | -0.01 | 0 | 100 | Europe - UK |
| Vinni (2023) - CD | MAIA-EA | 41 | -0.15 | 1 | 36.8 | Europe - Other |
| Vinni (2023) - UC | MAIA-EA | 16 | 0.4 | 1 | 62.5 | Europe - Other |
| Zamariola (2018) - Studies 4-6 | MAIA-EA | 263 | 0.02 | 0 | 77.9 | Europe - Other |
| Brand (2022) | MAIA-ND | 614 | -0.24 | 0 | 66 | Europe - Other |
| Da Costa Silva (2022) | MAIA-ND | 308 | -0.17 | 0 | 61.4 | Europe - Other |
| Desdentado (2022) | MAIA-ND | 391 | -0.2 | 0 | 61.4 | Europe - Other |
| Edwards & Lowe (2021) | MAIA-ND | 230 | -0.14 | 0 | 51 | Europe - UK |
| Gaggero (2021) - IT | MAIA-ND | 325 | -0.16 | 0 | 68 | Europe - Other |
| Gaggero (2021) - US | MAIA-ND | 250 | -0.38 | 0 | 68 | North America |
| Gaggero (2021) - SG | MAIA-ND | 239 | -0.26 | 0 | 62.8 | Asia |
| Pink (2021) | MAIA-ND | 172 | -0.05 | 0 | 100 | Europe - UK |
| Vinni (2023) - CD | MAIA-ND | 41 | 0.08 | 1 | 36.8 | Europe - Other |
| Vinni (2023) - UC | MAIA-ND | 16 | -0.37 | 1 | 62.5 | Europe - Other |
| Zamariola (2018) - Studies 4-6 | MAIA-ND | 263 | -0.17 | 0 | 77.9 | Europe - Other |
| Brand (2022) | MAIA-Noticing | 614 | -0.17 | 0 | 66 | Europe - Other |
| Da Costa Silva (2022) | MAIA-Noticing | 308 | -0.16 | 0 | 61.4 | Europe - Other |
| Desdentado (2022) | MAIA-Noticing | 391 | -0.02 | 0 | 61.4 | Europe - Other |
| Edwards & Lowe (2021) | MAIA-Noticing | 230 | -0.13 | 0 | 51 | Europe - UK |
| Gaggero (2021) - IT | MAIA-Noticing | 325 | -0.09 | 0 | 68 | Europe - Other |
| Gaggero (2021) - US | MAIA-Noticing | 250 | -0.16 | 0 | 68 | North America |
| Gaggero (2021) - SG | MAIA-Noticing | 239 | -0.04 | 0 | 62.8 | Asia |
| Pink (2021) | MAIA-Noticing | 172 | 0 | 0 | 86.4 | Europe - UK |
| Vinni (2023) - CD | MAIA-Noticing | 41 | 0 | 1 | 36.8 | Europe - Other |
| Vinni (2023) - UC | MAIA-Noticing | 16 | 0.04 | 1 | 62.5 | Europe - Other |
| Zamariola (2018) - Studies 4-6 | MAIA-Noticing | 263 | -0.20 | 0 | 77.9 | Europe - Other |
| Brand (2022) | MAIA-NW | 614 | -0.35 | 0 | 66 | Europe - Other |
| Da Costa Silva (2022) | MAIA-NW | 308 | -0.18 | 0 | 61.4 | Europe - Other |
| Desdentado (2022) | MAIA-NW | 391 | -0.23 | 0 | 61.4 | Europe - Other |
| Edwards & Lowe (2021) | MAIA-NW | 230 | -0.02 | 0 | 51 | Europe - UK |
| Gaggero (2021) - IT | MAIA-NW | 325 | -0.3 | 0 | 68 | Europe - Other |
| Gaggero (2021) - US | MAIA-NW | 250 | -0.27 | 0 | 68 | North America |
| Gaggero (2021) - SG | MAIA-NW | 239 | -0.26 | 0 | 62.8 | Asia |
| Pink (2021) | MAIA-NW | 172 | -0.24 | 0 | 100 | Europe - UK |
| Vinni (2023) - CD | MAIA-NW | 41 | -0.35 | 1 | 36.8 | Europe - Other |
| Vinni (2023) - UC | MAIA-NW | 16 | -0.14 | 1 | 62.5 | Europe - Other |
| Zamariola (2018) - Studies 4-6 | MAIA-NW | 263 | -0.45 | 0 | 77.9 | Europe - Other |
| Brand (2022) | MAIA-SR | 614 | -0.33 | 0 | 66 | Europe - Other |
| Da Costa Silva (2022) | MAIA-SR | 308 | -0.27 | 0 | 61.4 | Europe - Other |
| Desdentado (2022) | MAIA-SR | 391 | -0.13 | 0 | 61.4 | Europe - Other |
| Edwards & Lowe (2021) | MAIA-SR | 230 | -0.11 | 0 | 51 | Europe - UK |
| Gaggero (2021) - IT | MAIA-SR | 325 | -0.28 | 0 | 68 | Europe - Other |
| Gaggero (2021) - US | MAIA-SR | 250 | -0.28 | 0 | 68 | North America |
| Gaggero (2021) - SG | MAIA-SR | 239 | -0.29 | 0 | 62.8 | Asia |
| Pink (2021) | MAIA-SR | 172 | -0.12 | 0 | 100 | Europe - UK |
| Vinni (2023) - CD | MAIA-SR | 41 | -0.38 | 1 | 36.8 | Europe - Other |
| Vinni (2023) - UC | MAIA-SR | 16 | 0.18 | 1 | 62.5 | Europe - Other |
| Zamariola (2018) - Studies 4-6 | MAIA-SR | 263 | -0.28 | 0 | 77.9 | Europe - Other |
| Berenguer (2023) - F | MAIA-Total | 152 | -0.31 | 0 | 100 | Europe - Other |
| Berenguer (2023) - M | MAIA-Total | 86 | -0.21 | 0 | 0 | Europe - Other |
| Da Costa Silva (2022) | MAIA-Total | 308 | -0.38 | 0 | 61.4 | Europe - Other |
| Gaggero (2021) - IT | MAIA-Total | 325 | -0.37 | 0 | 68 | Europe - Other |
| Gaggero (2021) - US | MAIA-Total | 250 | -0.34 | 0 | 68 | North America |
| Gaggero (2021) - SG | MAIA-Total | 239 | -0.33 | 0 | 62.8 | Asia |
| Brand (2022) | MAIA-Trusting | 614 | -0.45 | 0 | 66 | Europe - Other |
| Da Costa Silva (2022) | MAIA-Trusting | 308 | -0.45 | 0 | 61.4 | Europe - Other |
| Desdentado (2022) | MAIA-Trusting | 391 | -0.23 | 0 | 61.4 | Europe - Other |
| Edwards & Lowe (2021) | MAIA-Trusting | 230 | -0.1 | 0 | 51 | Europe - UK |
| Gaggero (2021) - IT | MAIA-Trusting | 325 | -0.4 | 0 | 68 | Europe - Other |
| Gaggero (2021) - US | MAIA-Trusting | 250 | -0.35 | 0 | 68 | North America |
| Gaggero (2021) - SG | MAIA-Trusting | 239 | -0.37 | 0 | 62.8 | Asia |
| Pink (2021) | MAIA-Trusting | 172 | -0.31 | 0 | 100 | Europe - UK |
| Vinni (2023) - CD | MAIA-Trusting | 41 | -0.36 | 1 | 36.8 | Europe - Other |
| Vinni (2023) - UC | MAIA-Trusting | 16 | 0.25 | 1 | 62.5 | Europe - Other |
| Zamariola (2018) - Studies 4-6 | MAIA-Trusting | 263 | -0.40 | 0 | 77.9 | Europe - Other |
