# Supplementary material for: A systematic review and meta-analysis of the relationship between subjective interoception and alexithymia: Implications for construct definitions and measurement
Source: PLoS One. 2024 Nov 7;19(11):e0310411. doi: 10.1371/journal.pone.0310411 (PMC11542822; doi:10.1371/journal.pone.0310411)
Supplement: S10 File — (PDF) [file pone.0310411.s010.pdf]

Covidence #	Title	Authors	Year	Journal	DOI	Study	Notes	Extractors	Extraction Date
#1	A cross-modal A cross-modal component of alexithymia and its relationship with performance in a social cognition task batterycomponent of alexithymia and its relationship with performance in a social cognition task battery	R√∂sch, Sarah A.; Puhlmann, Lara M. C.; Preckel, Katrin	2022	Journal of Affective Disorders	10.1016/j.jad.2021.11.012	R√∂sch 2022	Irrelevant - did not meet inclusion criteria at title and abstract screening		
#2	A meta-analysis on the relationship between interoceptive awareness and alexithymia: Distinguishing interoceptive accuracy and sensibility	Trevisan, Dominic A.; Altschuler, Melody R.; Bagdasarov, Armen; Carlos, Carter; Duan, Suqian; Hamo, Ester; Kala, Shashwat; McNair, Morgan L.; Parker, Termara; Stahl, Dylan; Winkelman, Tatiana; Zhou, Melissa; McPartland, James C.	2019	Journal of Abnormal Psychology	10.1037/abn0000454	Trevisan 2019	Irrelevant - did not meet inclusion criteria at title and abstract screening		
#4	A pilot study investigating changes in neural processing after mindfulness training in elite athletes	Haase, Lori; May, April C.; Falahpour, Maryam; Isakovic, Sara; Simmons, Alan N.; Hickman, Steven D.; Liu, Thomas T.; Paulus, Martin P.	2015	Frontiers in Behavioral Neuroscience		Haase 2015	Irrelevant - did not meet inclusion criteria at title and abstract screening		
#5	Adaptive and maladaptive bodily awareness: Distinguishing interoceptive sensibility and interoceptive attention from anxiety,Äêinduced somatization in autism and alexithymia	Trevisan, Dominic A.; Mehling, Wolf E.; McPartland, James C.	2021	Autism Research	10.1002/aur.2458	Trevisan 2021	Irrelevant - did not meet inclusion criteria at title and abstract screening		
#6	Alcohol use and interoception,ÄîA narrative review	Wi≈õniewski, Pawe≈Ç; Maurage, Pierre; Jakubczyk, Andrzej; Trucco, Elisa M.; Suszek, Hubert; Kopera, Maciej	2021	Progress in Neuro-Psychopharmacology & Biological Psychiatry	10.1016/j.pnpbp.2021.110397	Wi≈õniewski 2021	Irrelevant - did not meet inclusion criteria at title and abstract screening		
#7	Alexithymia and empathy predict changes in autonomic arousal during affective stimulation	Bogdanov, Volodymyr B.; Bogdanova, Olena V.; Gorlov, Dmytro S.; Gorgo, Yuriy P.; Dirckx, Joris J. J.; Makarchuk, Mykola Y.; Schoenen, Jean; Critchley, Hugo	2013	Cognitive and Behavioral Neurology	10.1097/WNN.0000000000000002	Bogdanov 2013	Irrelevant - did not meet inclusion criteria at title and abstract screening		
#10	Alteration of interoceptive sensitivity: Expanding the spectrum of behavioural disorders in amyotrophic lateral sclerosis	Moretta, Pasquale; Spisto, Myriam; Ausiello, Francesco Pio; Iodice, Rosa; De Lucia, Natascia; Santangelo, Gabriella; Trojano, Luigi; Salvatore, Elena; Dubbioso, Raffaele	2022	Neurological Sciences	10.1007/s10072-022-06231-4	Moretta 2022	Irrelevant - did not meet inclusion criteria at title and abstract screening		
#11	Altered interoception in patients with borderline personality disorder: A study using heartbeat-evoked potentials	Flasbeck, Vera; Popkirov, Stoyan; Ebert, Andreas; Br√ºne, Martin	2020	Borderline Personality Disorder and Emotion Dysregulation	10.1186/s40479-020-00139-1	Flasbeck 2020	Irrelevant - did not meet inclusion criteria at title and abstract screening		
#12	An analysis of conscious fear and automatic threat response in psychopathy	Lamoureux, Virginia Ann; Glenn, Andrea L.	2021	Personality Disorders: Theory, Research, and Treatment	10.1037/per0000406	Lamoureux 2021	Irrelevant - did not meet inclusion criteria at title and abstract screening		
#14	Clinical application of somatosensory amplification in psychosomatic medicine	Nakao, Mutsuhiro; Barsky, Arthur J.	2007	BioPsychoSocial Medicine	10.1186/1751-0759-1-17	Nakao 2007	Irrelevant - did not meet inclusion criteria at title and abstract screening		
#15	Demystifying alexithymia: An empirical approach and roadmap for remediation	Aaron, Rachel	2017	Dissertation Abstracts International: Section B: The		Aaron 2017	Irrelevant - did not meet inclusion criteria at title and abstract screening		

				Sciences and Engineering					
#19	Do psychosocial factors moderate the relation between testosterone and female sexual desire? The role of interoception, alexithymia, defense mechanisms, and relationship status	Costa, Rui Miguel; Oliveira, Gon√ßalo; Pestana, Jos√©; Costa, David; Oliveira, Rui F.	2019	Adaptive Human Behavior and Physiology	10.1007/s40750-018-0102-7	Costa 2019	Irrelevant - did not meet inclusion criteria at title and abstract screening		
#22	Female sweet-likers have enhanced cross-modal interoceptive abilities	Iatridi, Vasiliki; Quadt, Lisa; Hayes, John E.; Garfinkel, Sarah N.; Yeomans, Martin R.	2021	Appetite	10.1016/j.appet.2021.105290	Iatridi 2021	Irrelevant - did not meet inclusion criteria at title and abstract screening		
#25	Interoception and alexithymia are related to differences between the self-reported and the objectively measured physical activity in patients with chronic musculoskeletal pain	Shizuma, Hisaharu; Abe, Tetsuya; Kanbara, Kenji; Amaya, Yusaku; Mizuno, Yasuyuki; Saka-Kochi, Yukie; Fukunaga, Mikihiko	2021	Journal of Psychosomatic Research	10.1016/j.jpsychores.2020.110324	Shizuma 2021	Irrelevant - did not meet inclusion criteria at title and abstract screening		
#27	Interoceptive awareness in patients with functional neurological symptoms	Ricciardi, Lucia; Demartini, Benedetta; Crucianelli, Laura; Krah√©, Charlotte; Edwards, Mark J.; Fotopoulou, Aikaterini	2016	Biological Psychology	10.1016/j.biopsycho.2015.10.009	Ricciardi 2016	Irrelevant - did not meet inclusion criteria at title and abstract screening		
#28	Links among emotional awareness, somatic awareness and autonomic homeostatic processing	Kanbara, Kenji; Fukunaga, Mikihiko	2016	BioPsychoSocial Medicine	10.1186/s13030-016-0059-3	Kanbara 2016	Irrelevant - did not meet inclusion criteria at title and abstract screening		
#30	More than words can say: A multi-disciplinary consideration of the psychotherapeutic evaluation and treatment of alexithymia	Duquette, Patrice	2020	Frontiers in Psychiatry	10.3389/fpsyt.2020.00433	Duquette 2020	Irrelevant - did not meet inclusion criteria at title and abstract screening		
#31	Paradoxical somatic information processing for interoception and anxiety in alexithymia	Terasawa, Yuri; Oba, Kentaro; Motomura, Yuki; Katsunuma, Ruri; Murakami, Hiroki; Moriguchi, Yoshiya	2021	European Journal of Neuroscience	10.1111/ejn.15528	Terasawa 2021	Irrelevant - did not meet inclusion criteria at title and abstract screening		
#32	Prevalence of autistic traits in functional neurological disorder and relationship to alexithymia and psychiatric comorbidity	Cole, Richard H.; Elmalem, Michael S.; Petrochilos, Panayiota	2023	Journal of the Neurological Sciences	10.1016/j.jns.2023.120585	Cole 2023	Irrelevant - did not meet inclusion criteria at title and abstract screening		
#33	Psychiatric symptomatology and perception of family functioning in an eating disorder day program	Wisotsky, Willo	2004	Dissertation Abstracts International: Section B: The Sciences and Engineering		Wisotsky 2004	Irrelevant - did not meet inclusion criteria at title and abstract screening		
#37	Self-reported interoceptive deficits in eating disorders: A meta-analysis of studies using the eating disorder inventory	Jenkinson, Paul M.; Taylor, Lauren; Laws, Keith R.	2018	Journal of Psychosomatic Research	10.1016/j.jpsychores.2018.04.005	Jenkinson 2018	Irrelevant - did not meet inclusion criteria at title and abstract screening		
#38	Sense of body ownership in patients affected by functional motor symptoms (conversion disorder)	Demartini, Benedetta; Ricciardi, Lucia; Crucianelli, Laura; Fotopoulou, Aikaterini; Edwards, Mark J.	2016	Consciousness and Cognition: An International Journal	10.1016/j.concog.2015.11.005	Demartini 2016	Irrelevant - did not meet inclusion criteria at title and abstract screening		
#39	The ability to understand emotions is associated with interoception‚Äêrelated insular activation and white matter integrity during aging	Dobrushina, Olga R.; Arina, Galina A.; Dobrynina, Larisa A.; Suslina, Anastasia D.; Solodchik, Polina O.; Belopasova, Anastasia V.; Gubanova, Mariia V.; Sergeeva, Anastasia N.; Kremneva, Elena I.; Krotenkova, Marina V.	2020	Psychophysiology	10.1111/psyp.13537	Dobrushina 2020	Irrelevant - did not meet inclusion criteria at title and abstract screening		

#59	Relationship between interoception and emotion regulation: New evidence from mixed methods.	Zamariola G; Frost N; Van Oost A; Corneille O; Luminet O	2019	J Affect Disord	10.1016/j.jad.2018.12.101	Zamariola 2019	Irrelevant - did not meet inclusion criteria at title and abstract screening		
#62	Atypical bodily self-awareness in vicarious pain responders.	Bowling NC; Botan V; Santiesteban I; Ward J; Banissy MJ	2019	Philos Trans R Soc Lond B Biol Sci	10.1098/rstb.2018.0361	Bowling 2019	Irrelevant - did not meet inclusion criteria at title and abstract screening		
#63	Neural and Behavioral Correlates of Impaired Insight and Self-Awareness in Substance Use Disorder.	Maracic CE; Moeller SJ	2021	Curr Behav Neurosci Rep	10.1007/s40473-021-00240-x	Maracic 2021	Irrelevant - did not meet inclusion criteria at title and abstract screening		
#67	The Anticipation and Perception of Affective Touch in Women with and Recovered from Anorexia Nervosa.	Crucianelli L; Demartini B; Goeta D; Nistic√≤ V; Saramandi A; Bertelli S; Todisco P; Gambini O; Fotopoulou A	2021	Neuroscience	10.1016/j.neuroscience.2020.09.013	Crucianelli 2021	Irrelevant - did not meet inclusion criteria at title and abstract screening		
#79	Oxytocin secretion is pulsatile in men and is related to social-emotional functioning.	Baskaran C; Plessow F; Silva L; Asanza E; Marengi D; Eddy KT; Sluss PM; Johnson ML; Misra M; Lawson EA	2017	Psychoneuroendocrinology	10.1016/j.psyneuen.2017.07.486	Baskaran 2017	Irrelevant - did not meet inclusion criteria at title and abstract screening		
#81	Effectiveness of a guided online mindfulness-focused intervention in a student population: Study protocol for a randomised control trial.	Schultchen D; K√ºchler AM; Schillings C; Weineck F; Karabatsiakis A; Ebert DD; Baumeister H; Pollatos O	2020	BMJ Open	10.1136/bmjopen-2019-032775	Schultchen 2020	Irrelevant - did not meet inclusion criteria at title and abstract screening		
#92	Interoceptive functioning in schizophrenia and schizotypy	Torregrossa, L.J.; Amedy, A.; Roig, J.; Prada, A.; Park, S.	2022	Schizophrenia Research	10.1016/j.schres.2021.11.046	Torregrossa 2022	Irrelevant - did not meet inclusion criteria at title and abstract screening		
#93	Interoceptive Abnormalities and Suicidality: A Systematic Review	Hielscher, E.; Zopf, R.	2021	Behavior Therapy	10.1016/j.beth.2021.02.012	Hielscher 2021	Irrelevant - did not meet inclusion criteria at title and abstract screening		
#118	Do interoceptive accuracy and interoceptive sensibility predict emotion regulation?	Schuette, SA; Zucker, NL; Smoski, MJ	2021	PSYCHOLOGICAL RESEARCH-PSYCHOLOGISCHE FORSCHUNG	10.1007/s00426-020-01369-2	Schuette 2021	Irrelevant - did not meet inclusion criteria at title and abstract screening		
#122	Association Between Interoception and Emotion Regulation in Individuals With Alcohol Use Disorder	Jakubczyk, A; Trucco, EM; Klimkiewicz, A; Skrzeszewski, J; Suszek, H; Zaorska, J; Nowakowska, M; Michalska, A; Wojnar, M; Kopera, M	2020	FRONTIERS IN PSYCHIATRY	10.3389/fpsyt.2019.01028	Jakubczyk 2020	Irrelevant - did not meet inclusion criteria at title and abstract screening		
#123	Exploring the role of interoception in autobiographical memory recollection	Messina, A; Basilico, S; Bottini, G; Salvato, G	2022	CONSCIOUSNESS AND COGNITION	10.1016/j.concog.2022.103358	Messina 2022	Irrelevant - did not meet inclusion criteria at title and abstract screening		
#124	Interoceptive accuracy is associated with emotional contagion in a valence- and sex-dependent manner	Lischke, A; Weippert, M; Mau-Moeller, A; Jacksteit, R; Pahnke, R	2020	SOCIAL NEUROSCIENCE	10.1080/17470919.2019.1690573	Lischke 2020	Irrelevant - did not meet inclusion criteria at title and abstract screening		
#125	Normal interoceptive accuracy in women with bulimia nervosa	Pollatos, O; Georgiou, E	2016	PSYCHIATRY RESEARCH	10.1016/j.psychres.2016.04.072	Pollatos 2016	Irrelevant - did not meet inclusion criteria at title and abstract screening		
#127	Sex-Specific Relationships Between Interoceptive Accuracy and Emotion Regulation	Lischke, A; Pahnke, R; Mau-Moeller, A; Jacksteit, R; Weippert, M	2020	FRONTIERS IN BEHAVIORAL NEUROSCIENCE	10.3389/fnbeh.2020.00067	Lischke 2020	Irrelevant - did not meet inclusion criteria at title and abstract screening		
#128	Direct and indirect effects of age on interoceptive accuracy and awareness across the adult lifespan	Murphy, J; Geary, H; Millgate, E; Catmur, C; Bird, G	2018	PSYCHONOMIC BULLETIN & REVIEW	10.3758/s13423-017-1339-z	Murphy 2018	Irrelevant - did not meet inclusion criteria at title and abstract screening		
#129	Interoceptive sensitivity, body image dissatisfaction, and body awareness in healthy individuals	Emanuelsen, L; Drew, R; Koteles, F	2015	SCANDINAVIAN JOURNAL OF PSYCHOLOGY	10.1111/sjop.12183	Emanuelsen 2015	Irrelevant - did not meet inclusion criteria at title and abstract screening		
#131	Autistic Traits Predict Underestimation of Emotional Abilities	Huggins, CF; Cameron, IM; Williams, JHG	2021	JOURNAL OF EXPERIMENTAL	10.1037/xge0000784	Huggins 2021	Irrelevant - did not meet inclusion criteria at title and abstract screening		

				PSYCHOLOGY-GENERAL					
#136	Investigating Multidimensional Interoceptive Awareness in a Japanese Population: Validation of the Japanese MAIA-J	Shoji, M; Mehling, WE; Hautzinger, M; Herbert, BM	2018	FRONTIERS IN PSYCHOLOGY	10.3389/fpsyg.2018.01855	Shoji 2018	Irrelevant - did not meet inclusion criteria at title and abstract screening		
#138	Differential changes in self-reported aspects of interoceptive awareness through 3 months of contemplative training	Bornemann, B; Herbert, BM; Mehling, WE; Singer, T	2015	FRONTIERS IN PSYCHOLOGY	10.3389/fpsyg.2014.01504	Bornemann 2015	Irrelevant - did not meet inclusion criteria at title and abstract screening		
#139	Psychometric Evaluation and Norms for the Multidimensional Assessment of Interoceptive Awareness (MAIA) in a Clinical Eating Disorders Sample	Brown, TA; Berner, LA; Jones, MD; Reilly, EE; Cusack, A; Anderson, LK; Kaye, WH; Wierenga, CE	2017	EUROPEAN EATING DISORDERS REVIEW	10.1002/erv.2532	Brown 2017	Irrelevant - did not meet inclusion criteria at title and abstract screening		
#141	Interoceptive sensitivity deficits in women recovered from bulimia nervosa	Klabunde, M; Acheson, DT; Boutelle, KN; Matthews, SC; Kaye, WH	2013	EATING BEHAVIORS	10.1016/j.eatbeh.2013.08.002	Klabunde 2013	Irrelevant - did not meet inclusion criteria at title and abstract screening		
#150	Body Attention, Ignorance and Awareness Scale: Assessing Relevant Concepts for Physical and Psychological Functioning in Psoriasis	Van Beugen, S; Ograczyk, A; Ferwerda, M; Smit, JV; Zeeuwen-Franssen, MEJ; Kroft, EBM; de Jong, EMGJ; Zalewska-Janowska, A; Donders, ART; van de Kerkhof, PCM; van Middendorp, H; Evers, AWM	2015	ACTA DERMATO-VENEREOLOGICA	10.2340/00015555-1977	VanBeugen 2015	Irrelevant - did not meet inclusion criteria at title and abstract screening		
#151	Interoceptive awareness is associated with acute alcohol-induced changes in subjective effects	Leganes-Fonteneau, M; Cheang, Y; Lam, Y; Garfinkel, S; Duka, T	2019	PHARMACOLOGY BIOCHEMISTRY AND BEHAVIOR	10.1016/j.pbb.2019.03.007	Leganes-Fonteneau 2019	Irrelevant - did not meet inclusion criteria at title and abstract screening		
#156	Hypermobile spectrum disorders symptoms in patients with functional neurological disorders and autism spectrum disorders: A preliminary study	Nistico, V; Iacono, A; Goeta, D; Tedesco, R; Giordano, B; Faggioli, R; Priori, A; Gambini, O; Demartini, B	2022	FRONTIERS IN PSYCHIATRY	10.3389/fpsyt.2022.943098	Nistico 2022	Irrelevant - did not meet inclusion criteria at title and abstract screening		
#157	Alexithymia in Adolescents with Autism Spectrum Disorder: Its Relationship to Internalising Difficulties, Sensory Modulation and Social Cognition	Milosavljevic, B; Leno, VC; Simonoff, E; Baird, G; Pickles, A; Jones, CRG; Erskine, C; Charman, T; Happe, F	2016	JOURNAL OF AUTISM AND DEVELOPMENTAL DISORDERS	10.1007/s10803-015-2670-8	Milosavljevic 2016	Irrelevant - did not meet inclusion criteria at title and abstract screening		
#158	Room to breathe: Using adaptive architecture to examine the relationship between alexithymia and interoception	Abdulhamid, H; Jager, N; Schnadelbach, H; Smith, AD	2022	JOURNAL OF PSYCHOSOMATIC RESEARCH	10.1016/j.jpsychores.2021.110708	Abdulhamid 2022	Irrelevant - did not meet inclusion criteria at title and abstract screening		
#160	Alexithymic traits can explain the association between puberty and symptoms of depression and anxiety in adolescent females	van der Cruijsen, R; Murphy, J; Bird, G	2019	PLOS ONE	10.1371/journal.pone.0210519	vanderCruijsen 2019	Irrelevant - did not meet inclusion criteria at title and abstract screening		
#162	A systematic review of how emotional self-awareness is defined and measured when comparing autistic and non-autistic groups	Huggins, CF; Donnan, G; Cameron, IM; Williams, JHG	2020	RESEARCH IN AUTISM SPECTRUM DISORDERS	10.1016/j.rasd.2020.101612	Huggins 2020	Irrelevant - did not meet inclusion criteria at title and abstract screening		
#165	How Do Fibromyalgia Patients With Alexithymia Experience Their Body? A Qualitative Approach	Calsius, J; Courtois, I; Stiers, J; De Bie, J	2015	SAGE OPEN	10.1177/2158244015574631	Calsius 2015	Irrelevant - did not meet inclusion criteria at title and abstract screening		
#167	Neuroimaging studies of alexithymia: physical, affective, and social perspectives	Moriguchi, Y; Komaki, G	2013	BIOPSYCHOSOCIAL MEDICINE	10.1186/1751-0759-7-8	Moriguchi 2013	Irrelevant - did not meet inclusion criteria at title and abstract screening		

#169	Interoceptive accuracy scores from the heartbeat counting task are problematic: Evidence from simple bivariate correlations	Zamariola, G; Maurage, P; Luminet, O; Corneille, O	2018	BIOLOGICAL PSYCHOLOGY	10.1016/j.biopsycho.2018.06.006	Zamariola 2018	Irrelevant - did not meet inclusion criteria at title and abstract screening		
#170	Interoceptive awareness and emotional eating in college women: the role of appetite and emotional awareness	Bullock, AJ; Goldbacher, EM	2021	JOURNAL OF AMERICAN COLLEGE HEALTH	10.1080/07448481.2021.1970566	Bullock 2021	Irrelevant - did not meet inclusion criteria at title and abstract screening		
#171	Selective Disruption of Sociocognitive Structural Brain Networks in Autism and Alexithymia	Bernhardt, BC; Valk, SL; Silani, G; Bird, G; Frith, U; Singer, T	2014	CEREBRAL CORTEX	10.1093/cercor/bht182	Bernhardt 2014	Irrelevant - did not meet inclusion criteria at title and abstract screening		
#172	Validation of an electronic version of the Self-Awareness Questionnaire in English and Italian healthy samples	Hughes, L; Betka, S; Longarzo, M	2019	INTERNATIONAL JOURNAL OF METHODS IN PSYCHIATRIC RESEARCH	10.1002/mpr.1758	Hughes 2019	Irrelevant - did not meet inclusion criteria at title and abstract screening		
#176	Levels of emotional awareness and autism: An fMRI study	Silani, G; Bird, G; Brindley, R; Singer, T; Frith, C; Frith, U	2008	SOCIAL NEUROSCIENCE	10.1080/17470910701577020	Silani 2008	Irrelevant - did not meet inclusion criteria at title and abstract screening		
#177	Personality and self-concept in subgroups of patients with anorexia nervosa and bulimia nervosa	Ciccolo, EBF; Johnsson, P	2002	SOCIAL BEHAVIOR AND PERSONALITY	10.2224/sbp.2002.30.4.347	Ciccolo 2002	Irrelevant - did not meet inclusion criteria at title and abstract screening		
#178	Extreme sensory processing patterns show a complex association with depression, and impulsivity, alexithymia, and hopelessness	Serafini, G; Gonda, X; Canepa, G; Pompili, M; Rihmer, Z; Amore, M; Engel-Yeger, B	2017	JOURNAL OF AFFECTIVE DISORDERS	10.1016/j.jad.2016.12.019	Serafini 2017	Irrelevant - did not meet inclusion criteria at title and abstract screening		
#179	DISTINCT INFLUENCE OF ANXIETY AND ALEXITHYMIA ON THE OBJECTIVE AND SUBJECTIVE ESTIMATES OF INDIVIDUAL EFFICACY IN HEARTBEAT DETECTION TASK	Dobrushina, O; Arina, G; Dobrynina, L; Belopasova, A; Gubanova, M; Suslina, A; Krotenkova, M	2020	PSYCHOPHYSIOLOGY		Dobrushina 2020	Irrelevant - did not meet inclusion criteria at title and abstract screening		
#181	Relationship of Alexithymia Ratings to Dopamine D2-type Receptors in Anterior Cingulate and Insula of Healthy Control Subjects but Not Methamphetamine-Dependent Individuals	Okita, K; Ghahremani, DG; Payer, DE; Robertson, CL; Mandelkern, MA; London, ED	2016	INTERNATIONAL JOURNAL OF NEUROPSYCHOPHARMACOLOGY	10.1093/ijnp/pyv129	Okita 2016	Irrelevant - did not meet inclusion criteria at title and abstract screening		
#182	Alexithymia as a risk factor for type 2 diabetes mellitus in the metabolic syndrome: a cross-sectional study	Lemche, AV; Chaban, OS; Lemche, E	2014	PSYCHIATRY RESEARCH	10.1016/j.psychres.2013.12.004	Lemche 2014	Irrelevant - did not meet inclusion criteria at title and abstract screening		
#183	Validity of the French form of the somatosensory amplification scale in a non-clinical sample	Bridou, M; Aguerre, C	2013	HEALTH PSYCHOLOGY RESEARCH	10.4082/hpr.2013.e11	Bridou 2013	Irrelevant - did not meet inclusion criteria at title and abstract screening		
#185	Psychometric validation and refinement of the Interoception Sensory Questionnaire (ISQ) in adolescents and adults on the autism spectrum	Suzman, E; Williams, ZJ; Feldman, JI; Failla, M; Cascio, CJ; Wallace, MT; Niarchou, M; Sutcliffe, JS; Wodka, E; Woynaroski, TG	2021	MOLECULAR AUTISM	10.1186/s13229-021-00440-y	Suzman 2021	Irrelevant - did not meet inclusion criteria at title and abstract screening		
#186	The Relevance of Interoception in Chronic Tinnitus: Analyzing Interoceptive Sensibility and Accuracy	Lau, P; Miesen, M; Wunderlich, R; Stein, A; Engell, A; Wollbrink, A; Gerlach, AL; Junghofer, M; Ehring, T; Pantev, C	2015	BIOMED RESEARCH INTERNATIONAL	10.1155/2015/487372	Lau 2015	Irrelevant - did not meet inclusion criteria at title and abstract screening		

#188	Alexithymic traits, independent of depression and anxiety, are associated with reduced sleep quality	Murphy, J; Wulff, K; Catmur, C; Bird, G	2018	PERSONALITY AND INDIVIDUAL DIFFERENCES	10.1016/j.paid.2018.03.023	Murphy 2018	Irrelevant - did not meet inclusion criteria at title and abstract screening		
#190	Different Aspects of Emotional Awareness in Relation to Motor Cognition and Autism Traits	Huggins, CF; Cameron, IM; Williams, JHG	2019	FRONTIERS IN PSYCHOLOGY	10.3389/fpsyg.2019.02439	Huggins 2019	Irrelevant - did not meet inclusion criteria at title and abstract screening		
#191	The Body in the Mind: On the Relationship Between Interoception and Embodiment	Herbert, BM; Pollatos, O	2012	TOPICS IN COGNITIVE SCIENCE	10.1111/j.1756-8765.2012.01189.x	Herbert 2012	Irrelevant - did not meet inclusion criteria at title and abstract screening		
#192	First-Hand Accounts of Interoceptive Difficulties in Autistic Adults	Trevisan, DA; Parker, T; McPartland, JC	2021	JOURNAL OF AUTISM AND DEVELOPMENTAL DISORDERS	10.1007/s10803-020-04811-x	Trevisan 2021	Irrelevant - did not meet inclusion criteria at title and abstract screening		
#193	Interoceptive sensibility predicts the ability to infer others' emotional states	Hubner, AM; Trempler, I; Gietmann, C; Schubotz, RI	2021	PLOS ONE	10.1371/journal.pone.0258089	Hubner 2021	Irrelevant - did not meet inclusion criteria at title and abstract screening		
#196	The Effects of a Standardized Cognitive-Behavioural Therapy and an Additional Mindfulness-Based Training on Interoceptive Abilities in a Depressed Cohort	Karanassios, G; Schultchen, D; Mohrle, M; Berberich, G; Pollatos, O	2021	BRAIN SCIENCES	10.3390/brainsci11101355	Karanassios 2021	Irrelevant - did not meet inclusion criteria at title and abstract screening		
#197	Knowledge of resting heart rate mediates the relationship between intelligence and the heartbeat counting task	Murphy, J; Millgate, E; Geary, H; Ichijo, E; Coll, MP; Brewer, R; Catmur, C; Bird, G	2018	BIOLOGICAL PSYCHOLOGY	10.1016/j.biopsycho.2018.01.012	Murphy 2018	Irrelevant - did not meet inclusion criteria at title and abstract screening		
#198	Multidimensional Interoception and Autistic Traits Across life Stages: Evidence From a Novel Eye-tracking Task	Yang, HX; Zhou, HY; Wei, Z; Wan, GB; Wang, Y; Wang, YY; Yang, TX; Lui, SSY; Chan, RCK	2022	JOURNAL OF AUTISM AND DEVELOPMENTAL DISORDERS	10.1007/s10803-021-05155-w	Yang 2022	Irrelevant - did not meet inclusion criteria at title and abstract screening		
#199	Individual differences in sensory and expectation driven interoceptive processes: a novel paradigm with implications for alexithymia, disordered eating and obesity	Young, HA; Gaylor, CM; de-Kerckhove, D; Benton, D	2021	SCIENTIFIC REPORTS	10.1038/s41598-021-89417-8	Young 2021	Irrelevant - did not meet inclusion criteria at title and abstract screening		
#200	Interoceptive Accuracy as a Function of Hypnotizability	Rosati, A; Belcari, I; Santarcangelo, EL; Sebastiani, L	2021	INTERNATIONAL JOURNAL OF CLINICAL AND EXPERIMENTAL HYPNOSIS	10.1080/00207144.2021.1954859	Rosati 2021	Irrelevant - did not meet inclusion criteria at title and abstract screening		
#202	Neural activity during interoceptive awareness and its associations with alexithymia-An fMRI study in major depressive disorder and non-psychiatric controls	Wiebking, C; Northoff, G	2015	FRONTIERS IN PSYCHOLOGY	10.3389/fpsyg.2015.00589	Wiebking 2015	Irrelevant - did not meet inclusion criteria at title and abstract screening		
#203	Tulsa 1000: a naturalistic study protocol for multilevel assessment and outcome prediction in a large psychiatric sample	Victor, TA; Khalsa, SS; Simmons, WK; Feinstein, JS; Sayitz, J; Aupperle, RL; Yeh, HW; Bodurka, J; Paulusl, MP	2018	BMJ OPEN	10.1136/bmjopen-2017-016620	Victor 2018	Irrelevant - did not meet inclusion criteria at title and abstract screening		
#204	A Bayesian computational model reveals a failure to adapt interoceptive precision estimates across depression, anxiety, eating, and substance use disorders	Tulsa 1000 Investigators; Smith, R; Kuplicki, R; Feinstein, J; Forthman, KL; Stewart, JL; Paulus, MP; Khalsa, SS	2020	PLOS COMPUTATIONAL BIOLOGY	10.1371/journal.pcbi.1008484	Tulsa1000Investigators 2020	Irrelevant - did not meet inclusion criteria at title and abstract screening		
#205	Body Conscious? Interoceptive Awareness, Measured by Heartbeat Perception, Is Negatively Correlated with Self-Objectification	Ainley, V; Tsakiris, M	2013	PLOS ONE	10.1371/journal.pone.0055568	Ainley 2013	Irrelevant - did not meet inclusion criteria at title and abstract screening		

#208	Interoceptive awareness mitigates deficits in emotional prosody recognition in Autism	Mulcahy, JS; Davies, M; Quadt, L; Critchley, HD; Garfinkel, SN	2019	BIOLOGICAL PSYCHOLOGY	10.1016/j.biopsycho.2019.05.011	Mulcahy 2019	Irrelevant - did not meet inclusion criteria at title and abstract screening		
#211	Evidence toward the potential absence of relationship between temporal and spatial heartbeats perception	Sophie, B; Marta, L; Marta, S; Joshua, K; Sarah, G; Hugo, C	2021	SCIENTIFIC REPORTS	10.1038/s41598-021-90334-z	Sophie 2021	Irrelevant - did not meet inclusion criteria at title and abstract screening		
#212	The relationship between heartbeat counting and heartbeat discrimination: A meta-analysis	Hickman, L; Seyedsalehi, A; Cook, JL; Bird, G; Murphy, J	2020	BIOLOGICAL PSYCHOLOGY	10.1016/j.biopsycho.2020.107949	Hickman 2020	Irrelevant - did not meet inclusion criteria at title and abstract screening		
#213	The Role of Interoception in the Pathogenesis and Treatment of Anorexia Nervosa: A Narrative Review	Jacquemot, AMMC; Park, R	2020	FRONTIERS IN PSYCHIATRY	10.3389/fpsyt.2020.00281	Jacquemot 2020	Irrelevant - did not meet inclusion criteria at title and abstract screening		
#214	Alexithymia in Multiple Sclerosis - Narrative Review	Grigorescu, C; Chalah, MA; Ayache, SS; Palm, U	2022	FORTSCHRITTE DER NEUROLOGIE PSYCHIATRIE	10.1055/a-1882-6544	Grigorescu 2022	Irrelevant - did not meet inclusion criteria at title and abstract screening		
#216	How Does Heartbeat Counting Task Performance Relate to Theoretically-Relevant Mental Health Outcomes? A Meta-Analysis	Desmedt, O; Van den Houte, M; Walentynowicz, M; Dekeyser, S; Luminet, O; Corneille, O	2022	COLLABRA-PSYCHOLOGY	10.1525/collabra.33271	Desmedt 2022	Irrelevant - did not meet inclusion criteria at title and abstract screening		
#217	The bodily fundament of empathy: The role of action, nonaction-oriented, and interoceptive body representations	Raimo, S; Boccia, M; Gaita, M; Canino, S; Torchia, V; Vetere, MA; Di Vita, A; Palermo, L	2022	PSYCHONOMIC BULLETIN & REVIEW	10.3758/s13423-022-02231-9	Raimo 2022	Irrelevant - did not meet inclusion criteria at title and abstract screening		
#218	Dissociation between Emotional Remapping of Fear and Disgust in Alexithymia	Scarpazza, C; Ladavas, E; di Pellegrino, G	2015	PLOS ONE	10.1371/journal.pone.0140229	Scarpazza 2015	Irrelevant - did not meet inclusion criteria at title and abstract screening		
#219	Does Long-Term Training in a Water Immersion Environment Change Interoception?	Baba, Y; Sato, D; Otsuru, N; Ikarashi, K; Fujimoto, T; Yamashiro, K	2021	INTERNATIONAL JOURNAL OF ENVIRONMENTAL RESEARCH AND PUBLIC HEALTH	10.3390/ijerph181910259	Baba 2021	Irrelevant - did not meet inclusion criteria at title and abstract screening		
#220	Ideal cardiovascular health in adolescents and young adults is associated with alexithymia over two decades later: Findings from the cardiovascular risk in Young Finns Study Department: Research Centre of Applied and Preventive Cardiovascular Medicine, Un	Karukivi, M; Jula, A; Pulkki-Raback, L; Hutri-Kahonen, N; Laitinen, TT; Viikari, J; Juonala, M; Raitakari, O	2020	PSYCHIATRY RESEARCH	10.1016/j.psychres.2020.112976	Karukivi 2020	Irrelevant - did not meet inclusion criteria at title and abstract screening		
#221	It matters what you practice: differential training effects on subjective experience, behavior, brain and body in the ReSource Project	Singer, T; Engert, V	2019	CURRENT OPINION IN PSYCHOLOGY	10.1016/j.copsyc.2018.12.005	Singer 2019	Irrelevant - did not meet inclusion criteria at title and abstract screening		
#222	Heart rate variability and its neural correlates during emotional face processing in social anxiety disorder	Gaebler, M; Daniels, JK; Lamke, JP; Fydrich, T; Walter, H	2013	BIOLOGICAL PSYCHOLOGY	10.1016/j.biopsycho.2013.06.009	Gaebler 2013	Irrelevant - did not meet inclusion criteria at title and abstract screening		
#223	Interoception and Its Interaction with Self, Other, and Emotion Processing: Implications for the Understanding of Psychosocial Deficits in Borderline Personality Disorder	Loffler, A; Foell, J; Bekrater-Bodmann, R	2018	CURRENT PSYCHIATRY REPORTS	10.1007/s11920-018-0890-2	Loffler 2018	Irrelevant - did not meet inclusion criteria at title and abstract screening		

#224	A heartbeat away from a valid tracking task. An empirical comparison of the mental and the motor tracking task	Kormendi, J; Ferentzi, E; Koteles, F	2022	BIOLOGICAL PSYCHOLOGY	10.1016/j.biopsycho.2022.108328	Kormendi 2022	Irrelevant - did not meet inclusion criteria at title and abstract screening		
#225	Reduced differentiation of emotion-associated bodily sensations in autism	Palser, ER; Galvez-Pol, A; Palmer, CE; Hannah, R; Fotopoulou, A; Pellicano, E; Kilner, JM	2021	AUTISM	10.1177/1362361320987950	Palser 2021	Irrelevant - did not meet inclusion criteria at title and abstract screening		
#226	The role of interoception in understanding others' affect. Dissociation between superficial and detailed appraisal of facial expressions	Dirupo, G; Corradi-Dell'Acqua, C; Kashef, M; Debbane, M; Badoud, D	2020	CORTEX	10.1016/j.cortex.2020.05.010	Dirupo 2020	Irrelevant - did not meet inclusion criteria at title and abstract screening		
#227	Know thyself: Exploring interoceptive sensitivity in Parkinson's disease	Ricciardi, L; Ferrazzano, G; Demartini, B; Morgante, F; Erro, R; Ganos, C; Bhatia, KP; Berardelli, A; Edwards, M	2016	JOURNAL OF THE NEUROLOGICAL SCIENCES	10.1016/j.jns.2016.03.019	Ricciardi 2016	Irrelevant - did not meet inclusion criteria at title and abstract screening		
#228	Social Bodies: Preliminary Evidence That Awareness of Embodied Emotions Is Associated With Recognition of Emotions in the Bodily Cues of Others	Blain, SD; Snodgress, MA; Nummenmaa, L; Peterman, JS; Glerean, E; Park, S	2023	PSYCHOLOGY OF CONSCIOUSNESS-THEORY RESEARCH AND PRACTICE	10.1037/cns0000352	Blain 2023	Irrelevant - did not meet inclusion criteria at title and abstract screening		
#230	Theory of Motivated Cue-Integration and COVID-19: Between Interoception, Somatization, and Radicalization	Shalev, I	2021	FRONTIERS IN PSYCHIATRY	10.3389/fpsyt.2021.631758	Shalev 2021	Irrelevant - did not meet inclusion criteria at title and abstract screening		
#231	Relationship between affective state and empathy in medical and psychology students	Bohler, TE; Brown, RF; Dunn, S	2021	AUSTRALIAN PSYCHOLOGIST	10.1080/00050067.2021.1926218	Bohler 2021	Irrelevant - did not meet inclusion criteria at title and abstract screening		
#232	The effect of aerobic exercise on interoception and cognitive function in healthy university students: a non-randomized controlled trial	Amaya, Y; Abe, T; Kanbara, K; Shizuma, H; Akiyama, Y; Fukunaga, M	2021	BMC SPORTS SCIENCE MEDICINE AND REHABILITATION	10.1186/s13102-021-00332-x	Amaya 2021	Irrelevant - did not meet inclusion criteria at title and abstract screening		
#235	Alexithymia, reward sensitivity and excessive exercise in non-binge-eaters versus severe binge eaters: Implications for primary and secondary exercise dependence	Lyvers, Michael; Truncali, Joseph; Stapleton, Peta; Thorberg, Fred Arne	2022	Current Psychology	10.1007/s12144-022-03511-2	Lyvers 2022	Irrelevant - did not meet inclusion criteria at title and abstract screening		
#238	Alexithymia, a compounding factor for eating and social avoidance symptoms in anorexia nervosa	Courty, Annaig; Godart, Nathalie; Lalanne, Christophe; Berthoz, Sylvie	2015	Comprehensive Psychiatry	https://doi.org/10.1016/j.comppsych.2014.09.011	Courty 2015	Irrelevant - did not meet inclusion criteria at title and abstract screening		
#246	Alexithymia, embodiment of emotions and interoceptive abilities	Scarpazza, Cristina; di Pellegrino, Giuseppe	2018	Current developments in alexithymia: A cognitive and affective deficit		Scarpazza 2018	Irrelevant - did not meet inclusion criteria at title and abstract screening		
#247	Individuals with autistic traits exhibit heightened alexithymia but intact interoceptive-exteroceptive sensory integration	Yang, Han-Xue; Zhou, Han-Yu; Zheng, Hong; Wang, Yi; Wang, Yan-Yu; Lui, Simon SY; Chan, Raymond CK	2021	Journal of Autism and Developmental Disorders		Yang 2021	Irrelevant - did not meet inclusion criteria at title and abstract screening		
#249	Interoceptive reliance as a major determinant of emotional eating in adult obesity	Willem, Cl√©mence; Nandrino, Jean-Louis; Doba, Karyn; Roussel, M√©line; Triquet, Claire; Verkindt, H√©l√®ne; Pattou, Fran√ßois; Gandolphe, Marie-Charlotte	2021	Journal of Health Psychology		Willem 2021	Irrelevant - did not meet inclusion criteria at title and abstract screening		
#257	Interoceptive abilities in inflammatory bowel diseases and irritable bowel syndrome	Fournier, Alicia; Mondillon, Laurie; Luminet, Olivier; Canini, Fr√©deric; Mathieu, Nicolas; Gauchez, Anne Sophie; Dantzer,	2020	Frontiers in psychiatry		Fournier 2020	Irrelevant - did not meet inclusion criteria at title and abstract screening		

		C√©cile; Bonaz, Bruno; Pellissier, Sonia							
#258	Do we need to accurately perceive our heartbeats? Cardioceptive accuracy and sensibility are independent from indicators of negative affectivity, body awareness, body image dissatisfaction, and alexithymia	K√∂rmendi, J√°nos; Ferentzi, Eszter; Petzke, Tara; G√°l, Vera; K√∂teles, Ferenc	202 3	Plos one		K√∂rmendi 2023	Irrelevant - did not meet inclusion criteria at title and abstract screening		
#260	On the embodiment of social cognition skills: The inner and outer body processing differently contributes to the affective and cognitive theory of mind	Canino, Silvia; Raimo, Simona; Boccia, Maddalena; Di Vita, Antonella; Palermo, Liana	202 2	Brain Sciences		Canino 2022	Irrelevant - did not meet inclusion criteria at title and abstract screening		
#261	Multidimensional assessment of interoceptive abilities, emotion processing and the role of early life stress in inflammatory bowel diseases	Atanasova, Konstantina; Lotter, Tobias; Reindl, Wolfgang; Lis, Stefanie	202 1	Frontiers in Psychiatry		Atanasova 2021	Irrelevant - did not meet inclusion criteria at title and abstract screening		
#268	Is It a Gut Feeling? Bodily Sensations Associated With the Experience of Valence and Arousal in Patients With Inflammatory Bowel Disease	Atanasova, Konstantina; Lotter, Tobias; Bekrater-Bodmann, Robin; Kleindienst, Nikolaus; Reindl, Wolfgang; Lis, Stefanie	202 2	Frontiers in Psychiatry		Atanasova 2022	Irrelevant - did not meet inclusion criteria at title and abstract screening		
#270	Interoceptive attention facilitates emotion regulation strategy use	Tan, Yafei; Wang, Xiaoqin; Blain, Scott D; Jia, Lei; Qiu, Jiang	202 3	International Journal of Clinical and Health Psychology		Tan 2023	Irrelevant - did not meet inclusion criteria at title and abstract screening		
#271	Individual differences in the evolution of mental health: Which are the most relevant in chronic pain?	Ribera, C Suso; Jornet-Gibert, M; Guerrero, L Camacho; Canudas, MV Ribera; Gallardo-Pujol, D	201 4	Abstracts/Personality and Individual Differences		Ribera 2014	Irrelevant - did not meet inclusion criteria at title and abstract screening		
#272	Dissociations between self-reported interoceptive accuracy and attention: Evidence from the Interoceptive Attention Scale	Gabriele, Eleonora; Spooner, Ria; Brewer, Rebecca; Murphy, Jennifer	202 2	Biological psychology		Gabriele 2022	Irrelevant - did not meet inclusion criteria at title and abstract screening		
#273	Losing trust in body sensations: Interoceptive awareness and depression symptom severity among primary care patients	Dunne, Julie; Flores, Michael; Gawande, Richa; Schuman-Olivier, Zev	202 1	Journal of affective disorders		Dunne 2021	Irrelevant - did not meet inclusion criteria at title and abstract screening		
#274	Difficulties in emotion regulation and deficits in interoceptive awareness in moderate and severe obesity	Willem, Cl√©mence; Gandolphe, Marie-Charlotte; Roussel, M√©line; Verkindt, H√©l√®ne; Pattou, Fran√ßois; Nandrino, Jean-Louis	201 9	Eating and Weight Disorders-Studies on Anorexia, Bulimia and Obesity		Willem 2019	Irrelevant - did not meet inclusion criteria at title and abstract screening		
#276	Functional (psychogenic non-epileptic/dissociative) seizures: why and how?	Ertan, Deniz; Aybek, Selma; LaFrance Jr, W Curt; Kanemoto, Kousuke; Tarrada, Alexis; Maillard, Louis; El-Hage, Wissam; Hingray, Coraline	202 2	Journal of Neurology, Neurosurgery & Psychiatry		Ertan 2022	Irrelevant - did not meet inclusion criteria at title and abstract screening		
#277	A feeling difficult to identify: Alexithymia is inversely associated with positive body image in adults from the United Kingdom	Longhurst, Phaedra; Swami, Viren	202 3	Journal of affective disorders		Longhurst 2023	Irrelevant - did not meet inclusion criteria at title and abstract screening		
#278	Battered Body, Battered Self: A Cross-Sectional Study of the Embodiment-Related Impairments of Female Victims of Intimate Partner Violence	Machorrinho, Joana; Veiga, Guida; Santos, Gra√ßa; Marmeleira, Jos√©	202 3	Journal of Aggression, Maltreatment & Trauma		Machorrinho 2023	Irrelevant - did not meet inclusion criteria at title and abstract screening		

#279	Emotional and cognitive modulation of cybersickness: The role of pain catastrophizing and body awareness	Mittelst√§dt, Justin Maximilian; Wacker, Jan; Stelling, Dirk	2019	Human Factors		Mittelst√§dt 2019	Irrelevant - did not meet inclusion criteria at title and abstract screening		
#280	Development of a Scale to Examine Responses to Bodily Sensations	Roche-Freedman, Katherine E; Brown, Rhonda F; Monaghan, Conal; Thorsteinsson, Einar; Brown, John	2022	Psychological Reports		Roche-Freedman 2022	Irrelevant - did not meet inclusion criteria at title and abstract screening		
#281	Coping and beliefs as predictors of functioning and psychological adjustment in fibromyalgia subgroups	Rubio Fidel, Laura; Garc√≠a-Palacios, Azucena; Herrero, Roc√≠o; Molinari, Guadalupe; Suso-Ribera, Carlos	2022	Pain Research and Management		RubioFidel 2022	Irrelevant - did not meet inclusion criteria at title and abstract screening		
#282	The differential relationship between self-reported interoceptive accuracy and attention with psychopathology	Brand, Sebastian; Petzke, Tara M; Withh√∂ft, Michael	2022	Zeitschrift f√ºr Klinische Psychologie und Psychotherapie		Brand 2022	Irrelevant - did not meet inclusion criteria at title and abstract screening		
#284	Alexithymia is associated with a multidomain, multidimensional failure of interoception: Evidence from novel tests.	Murphy, Jennifer; Catmur, Caroline; Bird, Geoffrey	2018	Journal of Experimental Psychology: General		Murphy 2018	Irrelevant - did not meet inclusion criteria at title and abstract screening		
#286	Interoceptive accuracy and body awareness‚ÄìTemporal and longitudinal associations in a non-clinical sample	Ferentzi, Eszter; Drew, Raechel; Tihanyi, Benedek T; K√∂teles, Ferenc	2018	Physiology & behavior		Ferentzi 2018	Irrelevant - did not meet inclusion criteria at title and abstract screening		
#3	A network analysis of interoception, self-awareness, empathy, alexithymia, and autistic traits	Yang, Han-Xue; Hu, Hui-Xin; Zhang, Yi-Jing; Wang, Yi; Lui, Simon S. Y.; Chan, Raymond C. K.	2022	European Archives of Psychiatry and Clinical Neuroscience	10.1007/s00406-021-01274-8	Yang 2022	Exclusion reason: No reported correlation;		
#16	Disentangling interoceptive abilities in alexithymia	Scarpazza, Cristina; Zangrossi, Andrea; Huang, Yu-Chun; Sartori, Giuseppe; Massaro, Sebastiano	2021	Psychological Research	10.1007/s00426-021-01538-x	Scarpazza 2021	Exclusion reason: Duplicate;		
#20	Exploring the cognitive, emotional and sensory correlates of social anxiety in autistic and neurotypical adolescents	Pickard, Hannah; Hirsch, Colette; Simonoff, Emily; Happ√©, Francesca	2020	Journal of Child Psychology and Psychiatry	10.1111/jcpp.13214	Pickard 2020	Exclusion reason: Paediatric population;		
#26	Interoception in anorexia nervosa: Exploring associations with alexithymia and autistic traits	Kinnaird, Emma; Stewart, Catherine; Tchanturia, Kate	2020	Frontiers in Psychiatry	10.3389/fpsyt.2020.00064	Kinnaird 2020	Exclusion reason: No interoceptive scale;		
#34	Quadratic relationship between alexithymia and interoceptive accuracy, and results from a pilot mindfulness intervention	Aaron, Rachel V.; Blain, Scott D.; Snodgress, Matthew A.; Park, Sohee	2020	Frontiers in Psychiatry	10.3389/fpsyt.2020.00132	Aaron 2020	Exclusion reason: No interoceptive scale;		
#36	Relationship between interoceptive sensibility and somatoform disorders in adults with autism spectrum traits The mediating role of alexithymia and emotional dysregulation	Zdankiewicz-≈öciga≈Ça, El≈ºbieta; ≈öciga≈Ça, Dawid; Sikora, Joanna; Kwaterniak, Wanda; Longobardi, Claudio	2021	PLoS ONE	10.1371/journal.pone.0255460	Zdankiewicz-≈öciga≈Ça 2021	Exclusion reason: Relationship between interoception and alexithymia not investigated;		
#40	The effect of a single yoga class on interoceptive accuracy in patients affected by anorexia nervosa and in healthy controls: A pilot study	Demartini, Benedetta; Goeta, Diana; Marchetti, Mattia; Bertelli, Sara; Anselmetti, Simona; Cocchi, Alessandra; Ischia, Maddalena; Gambini, Orsola	2021	Eating and Weight Disorders	10.1007/s40519-020-00950-3	Demartini 2021	Exclusion reason: No interoceptive scale;		
#41	The psychophysiological mechanisms of alexithymia in autism spectrum disorder	Gaigg, Sebastian B.; Cornell, Anna S. F.; Bird, Geoffrey	2018	Autism	10.1177/1362361316667062	Gaigg 2018	Exclusion reason: No interoceptive scale;		
#50	Relationships between alexithymia, interoception, and emotional empathy in autism spectrum disorder.	Butera CD; Harrison L; Kilroy E; Jayashankar A; Shipkova M; Pruyser A; Aziz-Zadeh L	2023	Autism	10.1177/13623613221111310	Butera 2023	Exclusion reason: Paediatric population;		

#58	Importance of considering interoceptive abilities in alexithymia assessment.	Fournier A; Luminet O; Dambrun M; Dutheil F; Pellissier S; Mondillon L	2019	PeerJ	10.7717/peerj.7615	Fournier 2019	Exclusion reason: No interoceptive scale;		
#70	Discrepancies between dimensions of interoception in autism: Implications for emotion and anxiety.	Garfinkel SN; Tiley C; O'Keeffe S; Harrison NA; Seth AK; Critchley HD	2016	Biol Psychol	10.1016/j.biopsycho.2015.12.003	Garfinkel 2016	Exclusion reason: Relationship between interoception and alexithymia not investigated;		
#78	[Interoceptive difficulties in children and adolescents with severe form of somatic symptom disorder: A pilot study with nineteen participants].	Heniquez A; Lahaye H; Boissel L; Guil√© JM; Benarous X	2022	Encephale	10.1016/j.encep.2022.06.003	Heniquez 2022	Exclusion reason: Paediatric population;		
#242	Individual differences in emotional processing and autobiographical memory: interoceptive awareness and alexithymia in the fading affect bias	Muir, Kate; Madill, Anna; Brown, Charity	2017	Cognition and Emotion	10.1080/02699931.2016.1225005	Muir 2017	Exclusion reason: Relationship between interoception and alexithymia not investigated;		
#255	Bodily self-consciousness in Autism Spectrum Disorder: investigating the relationship between interoception, self-representation and empathy	Mul, Cari-lene	2019			Mul 2019	Exclusion reason: Duplicate;		
#275	No effect of age on emotion recognition after accounting for cognitive factors and depression	Murphy, Jennifer; Millgate, Edward; Geary, Hayley; Catmur, Caroline; Bird, Geoffrey	2019	Quarterly Journal of Experimental Psychology		Murphy 2019	Exclusion reason: Relationship between interoception and alexithymia not investigated;		
#283	INTEROCEPTIVE ATTENTION AND ACCURACY	T√ºnte, Markus R; Petzke, Tara M; Brand, Sebastian; Murphy, Jennifer; Witth√∂ft, Michael; Hoehl, Stefanie; Weymar, Mathias; Ventura-Bort, Carlos					Exclusion reason: Duplicate;		
#8	Alexithymia and sensory processing sensitivity: Areas of overlap and links to sensory processing styles	Jakobson, Lorna S.; Rigby, Sarah N.	2021	Frontiers in Psychology	10.3389/fpsyg.2021.583786	Jakobson 2021	Included	KVB, JK	2/11/2023
#9	Alexithymia mediates the relationship between interoceptive sensibility and anxiety	Palser, Eleanor R.; Palmer, Clare E.; Galvez-Pol, Alejandro; Hannah, Ricci; Fotopoulou, Aikaterini; Kilner, James M.	2018	PLoS ONE	10.1371/journal.pone.0203212	Palser 2018	Excluded - did not report correlations		
#13	Clarifying the relationship between alexithymia and subjective interoception	Gaggero, Giulia; Bizzego, Andrea; Dellantonio, Sara; Pastore, Luigi; Lim, Mengyu; Esposito, Gianluca	2021	PLoS ONE	10.1371/journal.pone.0261126	Gaggero 2021	Included	KVB, JK	2/11/2023
#17	Disentangling the role of interoceptive sensibility in alexithymia, emotion dysregulation, and depression in healthy individuals	Desdentado, Lorena; Miragall, Marta; Llorens, Roberto; Ba√±os, Rosa Mar√≠a	2022	10.1007/s12144-022-03153-4	Desdentado 2022	Included	KVB, JK	2/11/2023	
#18	Do alexithymia and negative affect predict poor sleep quality? The moderating role of interoceptive sensibility	Huang, Yun-Hsin; Yang, Chien-Ming; Huang, Ya-Chuan; Huang, Yu-Ting; Yen, Nai-Shing	2022	PLoS ONE	10.1371/journal.pone.0275359	Huang 2022	Included	KVB, JK	2/11/2023
#21	Exploring three levels of interoception in people with functional motor disorders	Ricciardi, Lucia; Nistic√≤, Veronica; Andrenelli, Elisa; Cunha, Joana Macedo; Demartini, Benedetta; Kirsch, Louise P.; Crucianelli, Laura; Yogarajah, Mahinda; Morgante, Francesca; Fotopoulou, Aikaterini; Edwards, Mark J.	2021	Parkinsonism & Related Disorders	10.1016/j.parkreldis.2021.03.029	Ricciardi 2021	Included	KVB, JK	1/3/2024

#23	How do self‚Äêassessment of alexithymia and sensitivity to bodily sensations relate to alcohol consumption?	Betka, Sophie; Pfeifer, Gaby; Garfinkel, Sarah; Prins, Hielke; Bond, Rod; Sequeira, Henrique; Duka, Theodora; Critchley, Hugo	2018	Alcoholism: Clinical and Experimental Research	10.1111/acer.13542	Betka 2018	Included	KVB, JK	1/3/2024
#29	Manipulating the sensation of feeling fat: The role of alexithymia, interoceptive sensibility and perfectionism	Pink, Aimee E.; Williams, Claire; Lee, Michelle; Young, Hayley A.; Harrison, Sophie; Davies, Amy Eldred; Price, Menna	2021	Physiology & Behavior	10.1016/j.physbeh.2021.113501	Pink 2021	Included	KVB, JK	15/11/2023
#35	Relationship between interoceptive accuracy, interoceptive sensibility, and alexithymia	Zamariola, Giorgia; Vlemincx, Elke; Corneille, Olivier; Luminet, Olivier	2018	Personality and Individual Differences	10.1016/j.paid.2017.12.024	Zamariola 2018	Included	KVB, JK	
#43	Disentangling interoceptive abilities in alexithymia.	Scarpazza C; Zangrossi A; Huang YC; Sartori G; Massaro S	2022	Psychol Res	10.1007/s00426-021-01538-x	Scarpazza 2022	Excluded - did not report correlations		
#47	Interoceptive Sensibility, Alexithymia, and Emotion Regulation in Individuals Suffering from Fibromyalgia.	Schmitz N; Napieralski J; Schroeder D; Loeser J; Gerlach AL; Pohl A	2021	Psychopathology	10.1159/000513774	Schmitz 2021	Included	KVB, JK	
#57	Self-Reported Body Awareness: Validation of the Postural Awareness Scale and the Multidimensional Assessment of Interoceptive Awareness (Version 2) in a Non-clinical Adult French-Speaking Sample.	Da Costa Silva L; Belrose C; Trousselard M; Rea B; Seery E; Verdonk C; Duffaud AM	2022	Front Psychol	10.3389/fpsyg.2022.946271	DaCostaSilva 2022	Included	KVB, JK	
#97	Alexithymia and Alcohol Use: Evaluating the Role of Interoceptive Sensibility with the Revised Multidimensional Assessment of Interoceptive Awareness	Lyvers, M.; Thorberg, F.A.	2023	Journal of Psychopathology and Behavioral Assessment	10.1007/s10862-023-10034-y	Lyvers 2023	Included	KVB, JK	10/9/2023
#107	Testing the independence of self-reported interoceptive accuracy and attention	Murphy, J; Brewer, R; Plans, D; Khalsa, SS; Catmur, C; Bird, G	2020	QUARTERLY JOURNAL OF EXPERIMENTAL PSYCHOLOGY	10.1177/1747021819879826	Murphy 2020	Included	KVB, JK	1/3/2024
#114	A novel self-report scale of interoception: the three-domain interoceptive sensations questionnaire (THISQ)	Vlemincx, E; Walentynowicz, M; Zamariola, G; Van Oudenhove, L; Luminet, O	2021	PSYCHOLOGY & HEALTH	10.1080/08870446.2021.2009479	Vlemincx 2021	Included	KVB, JK	1/3/2024
#133	The relationships between interoception and alexithymic trait. The Self-Awareness Questionnarie in healthy subjects	Longarzo, M; D'Olimpio, F; Chiavazzo, A; Santangelo, G; Trojano, L; Grossi, D	2015	FRONTIERS IN PSYCHOLOGY	10.3389/fpsyg.2015.01149	Longarzo 2015	Included	KVB, JK	10/9/2023
#135	Exploring the contributions of affective constructs and interoceptive awareness to feeling fat	Morales, C; Dolan, SC; Anderson, DA; Anderson, LM; Reilly, EE	2022	EATING AND WEIGHT DISORDERS-STUDIES ON ANOREXIA BULIMIA AND OBESITY	10.1007/s40519-022-01490-8	Morales 2022	Included	KVB, JK	10/9/2023
#143	Alexithymia: a general deficit of interoception	Brewer, R; Cook, R; Bird, G	2016	ROYAL SOCIETY OPEN SCIENCE	10.1098/rsos.150664	Brewer 2016	Included	KVB, JK	13/9/2023
#164	The Role of Interoceptive Sensibility and Emotional Conceptualization for the Experience of Emotions	Ventura-Bort, C; Wendt, J; Weymar, M	2021	FRONTIERS IN PSYCHOLOGY	10.3389/fpsyg.2021.712418	Ventura-Bort 2021	Included	KVB, JK	13/9/2023
#184	Shared and unique interoceptive deficits in high alexithymia and neuroticism	Gaggero, G; Dellantonio, S; Pastore, L; Sng, KHL; Esposito, G	2022	PLOS ONE	10.1371/journal.pone.0273922	Gaggero 2022	Excluded - sample not independent to Gaggero 2021		
#233	The Feeling of Me Feeling for You: Interoception, Alexithymia and Empathy in Autism	Mul, Cari-l√®ne; Stagg, Steven D.; Herbelin, Bruno; Aspell, Jane E.	2018	Journal of Autism and Developmental Disorders	10.1007/s10803-018-3564-3	Mul 2018	Included	KVB, NG	2/9/2023

#237	Associations Between Mental Health, Interoception, Psychological Flexibility, and Self-as-Context, as Predictors for Alexithymia: A Deep Artificial Neural Network Approach.	Edwards DJ; Lowe R	2021	Front Psychol	10.3389/fpsyg.2021.637802	Edwards 2021	Included	KVB, NG	5/2/2024
#239	Ready, set, ‚Ä¶and difficultly slowing down: What role does alexithymia, emotional regulation and interoceptive awareness play in exercise dependence?	Sweetnam, Taylor J.; Flack, Mal	2023	Acta Psychologica	https://doi.org/10.1016/j.actpsy.2023.103958	Sweetnam 2023	Included	KVB, NG	2/11/2023
#240	Relationships between alexithymia and psychological characteristics associated with eating disorders	Taylor, Graeme J.; Parker, James D.A.; Bagby, R.Michael; Bourke, Michael P.	1996	Journal of Psychosomatic Research	https://doi.org/10.1016/S0022-3999(96)00224-3	Taylor 1996	Included	KVB, NG	2/11/2023
#241	Adult attachment styles and emotional regulation: The role of interoceptive awareness and alexithymia	Ferraro, Isabella K; Taylor, Amanda M	2021	Personality and Individual Differences	https://doi.org/10.1016/j.paid.2021.110641	Ferraro 2021	Included	KVB, NG	1/3/2024
#243	Interoceptive Awareness, Alexithymia, and Sexual Function	Berenguer, Cl√°udia; Reb√¥lo, Catarina; Costa, Rui Miguel	2019	Journal of Sex & Marital Therapy	10.1080/0092623X.2019.1610128	Berenguer 2019	Included	KVB, NG	1/3/2024
#248	Alexithymic characteristics and interoceptive abilities are associated with disease severity and levels of C-reactive protein and cytokines in patients with inflammatory bowel disease	Vinni, Eleni; Karaivazoglou, Katerina; Tourkochristou, Evanthia; Tsounis, Efthymios; Kalogeropoulou, Maria; Konstantopoulou, Georgia; Lourida, Theoni; Kafentzi, Theodora; Lampropoulou, Efi; Rodi, Maria	2023	Annals of Gastroenterology		Vinni 2023	Included	KVB, NG	15/11/2023
#262	Construct validity of the sensory profile interoception scale: Measuring sensory processing in everyday life	Dunn, Winnie; Brown, Catana; Breitmeyer, Angela; Salwei, Ashley	2022	Frontiers in Psychology		Dunn 2022	Included	KVB, NG	15/11/2023
#265	Untangling self-reported interoceptive attention and accuracy: Evidence from the european portuguese validation of the body perception questionnaire and the interoceptive accuracy scale	Campos, Carlos; Rocha, Nuno; Barbosa, Fernando	2021			Campos 2021	Included	KVB, NG	15/11/2023
#266	He Who Seeks Finds (Bodily Signals): Differential Effects of Self-Reported Interoceptive Attention and Accuracy on Subclinical Psychopathology in a German-Speaking Sample	T√ºnte, Markus R; Petzke, Tara; Brand, Sebastian; Murphy, Jennifer; Witth√∂ft, Michael; Hoehl, Stefanie; Weymar, Mathias; Ventura-Bort, Carlos	2022			T√ºnte 2022	Included	KVB, NG	15/11/2023
#285	Emotional Dysfunction and Interoceptive Challenges in Adults with Autism Spectrum Disorders	Bonete, Saray; Molinero, Clara; Ruisanchez, Daniela	2023	Behavioral Sciences		Bonete 2023	Included	KVB, NG	1/12/2023
#288	Examining the Incremental Validity of the Perth Alexithymia Questionnaire (PAQ) Relative to the 20-Item Toronto Alexithymia Scale (TAS-20)	Zahid, Aqsa; Taylor, Graeme J.; Lau, Sharlane C. L.; Stone, Suddene; Bagby, R. Michael	2023	Journal of Personality Assessment	10.1080/00223891.2023.2201831	Zahid 2023	Included	KVB, NG	5/2/2024
#289	Emotional regulation deficits in autism spectrum disorder: The role of alexithymia and interoception	Ben Hassen, Nour; Molins, Francisco; Garrote-Petisco, Dolores; Serrano, Miguel √Ångel	2023	Research in Developmental Disabilities	https://doi.org/10.1016/j.ridd.2022.104378	BenHassen 2023	Included	KVB, NG	1/12/2023

#290	The Interoception Sensory Questionnaire (ISQ): A Scale to Measure Interoceptive Challenges in Adults	Fiene, L.; Ireland, M. J.; Brownlow, C.	2018	Journal of Autism and Developmental Disorders	10.1007/s10803-018-3600-3	Fiene 2018	Included	KVB, NG	
#291	Bridging the Gap between Interoception and Mental Health: The German Validation of the Interoceptive Accuracy Scale (IAS) and its Relation to Psychopathological Symptom Burden	Brand, Sebastian; Meis, Annelie; T√ºnte, Markus; Murphy, Jennifer; Woller, Joshua Pepe; Jungmann, Stefanie; Witth√∂ft, Michael; Hoehl, Stefanie; Weymar, Mathias; Hermann, Christiane; Ventura-Bort, Carlos	2022		10.31234/osf.io/jak6b	Brand 2022	Included	KVB, NG	
#292	The association of interoceptive awareness and alexithymia with neurotransmitter concentrations in insula and anterior cingulate	Ernst, Jutta; B√∂ker, Heinz; H√§ttenschwiler, Joe; Sch√ºpbach, Daniel; Northoff, Georg; Seifritz, Erich; Grimm, Simone	2014	Social Cognitive and Affective Neuroscience	10.1093/scan/nst058	Ernst 2014	Included	KVB, NG	
