# Supplementary material for: A systematic review and meta-analysis of the relationship between subjective interoception and alexithymia: Implications for construct definitions and measurement
Source: PLoS One. 2024 Nov 7;19(11):e0310411. doi: 10.1371/journal.pone.0310411 (PMC11542822; doi:10.1371/journal.pone.0310411)
Supplement: Attachment — Submitted filename: Response to Reviewers.docx [file pone.0310411.s011.docx]

# **Response to Editor and Reviewers**

# **Editor’s Comments**

*From a methodological standpoint, there are no major issues to resolve, except for providing inter-rater reliability for study selection and data extraction, as recommended by several systematic review and meta-analysis guidelines.*

We confirm that inter-rater reliability for study selection, data extraction, and risk of bias have been provided. Due to the length of the manuscript, we chose to summarise these results in the main reporting, with full inter-rater reliability results reported in the revised Supporting Information (File S2).

*From a conceptual standpoint, there are some important topics that need to be clarified or discussed in the manuscript, particularly regarding the criteria for classifying questionnaires and how alternative classification approaches could also be feasible.*

We have provided clearer definitions for adaptive and maladaptive interoception in the Introduction (p. 2, L 62-73), which we feel better clarifies our reasoning for classifying questionnaires in this way. Whilst we believe that the suggestion to classify questionnaires according to the valence of assessed sensations is valuable, we feel that our classification provides a nuanced account for the reviewed measures, supported by existing and emerging interoceptive frameworks and their emotional outcomes following our meta-analytic findings. However, we have specified that our findings may provide a foundation for alternative, appropriate classification systems in future, such as the valence of sensations in self-reports.

*Lastly, despite the manuscript being extremely well-written, I believe there is still some room for improvement, particularly considering the complexity of the analysis and results. The reviewers provide several comments that can be useful for the authors to make the manuscript clearer to the reader (e.g., summary figure).*

We strongly agreed with this suggestion from the Reviewers. We have added a workflow diagram (Fig 4) and a summary figure of the meta-analysis correlations (heat map, Fig 5).

**Reviewers' comments**

## **Reviewer #1**

*This systematic review and these meta-analyses are excellent for several reasons. The authors greatly explained the actual state of the literature on self-report interoception and followed the most up-to-date recommendations in the field. The method is overall very good. The discussion is complete and insightful for the field. Once published, this review will have a huge and, more importantly, a very positive impact on the literature, as it promotes valid and nuanced interpretations of data. I have rarely read such a well-written manuscript. Many congrats to the authors!*

Many thanks to the reviewer for their kind and considered feedback on the paper, especially in terms of its impact on the field! It is greatly appreciated. We have provided our responses to each of their comments and suggestions below. *You can find below my minor comments:
1. Please report the inter-rater reliability for the selection of papers, their extraction, and their assessment.*

We concur that this is necessary information to report and apologise for the omission. Due to the length of this paper, we have provided a summary of agreements/reliability amongst reviewers in the paper (Results - Study Selection; Methods – Risk of bias assessment), with more detailed information regarding inter-rater reliability for study selection, data extraction, and risk of bias as a new Supporting Material (File S2). *2. There seems to be a typo in this sentence as the correlation between ICQ and alexithymia is reported twice with different results: “Meta-analysis demonstrated a strong positive association for the ICQ and alexithymia, r(9) = 0.57, p <.001, suggesting greater struggles to interpret non-affective interoceptive states coincides with difficulties identifying and describing feelings, and a preference for externally oriented thinking. A strong positive effect was further observed between the ICQ and alexithymia, r(2) = 0.53, p <.001, indicating that greater difficulty registering or interpreting interoceptive sensations relates to higher alexithymia.”*

Many thanks to the reviewer for identifying this typo! This has been amended to report results for the ICQ and **ISQ** in terms of their association with global alexithymia. (p. 24, L 653). *3. I do not agree that the THISQ is a measure of neutral interoceptive attention, as attentional aspects are not mentioned in the items of this questionnaire. Neutral interoceptive detection would be more relevant. Similarly, I don’t think that the BAQ is a measure of adaptive interoceptive attention. Items are more related to the capacity to notice and predict body reactions to internal and external factors such as weather, seasons, foods, blows, diseases, and energy level. In sum, the THISQ and the BAQ are questionnaires assessing the capacity to detect, notice, or predict bodily cues that should be detected/predicted for maintaining well-being or homeostasis (i.e., adaptive interoceptive abilities).*

We thank the reviewer for their insightful comments regarding the THISQ and BAQ and appreciate the opportunity to clarify our discussion of these measures. We acknowledge your point that the THISQ possibly assesses interoceptive detection. Indeed, Vlemincx and colleagues (2023, <https://www.tandfonline.com/doi/full/10.1080/08870446.2021.2009479>) characterise the THISQ as a measure of self-reported perception of neutral respiratory, cardiac, and gastroesophageal sensations. In their discussion of evidence for convergent validity, Vlemincx et al. further note that THISQ scores correlated moderately with other measures of non-emotional interoceptive attention, such as the BAQ and the Noticing scale of the MAIA. Considering the congruence between the authors’ and our interpretations, we feel that ‘neutral attention’ best characterises the THISQ, in accordance with current evidence. This information has been added to the Discussion (p. 44, L 1185). We have also provided further reasoning for what the authors of THISQ mean by neutral in its subsection within Administered Measures (p. 21, L 541-543), and hope this clarifies the logic behind classifying the questionnaire a measure of neutral attention.

The BAQ is currently classified as a self-report of interoceptive attention in the literature (e.g., Trevisan et al. 2020, <https://onlinelibrary.wiley.com/doi/10.1002/aur.2458>; Vlemincx et al. 2023), as noticing and frequency of awareness involves orienting attention to salient interoceptive signals (Mehling, 2012, <https://journals.plos.org/plosone/article?id=10.1371/journal.pone.0048230>), therein a key subfactor of interoceptive attention (Desmedt et al., 2023). Due to these factors, we are inclined to comply with current opinion and evidence supporting the BAQ’s classification as a measure of interoceptive attention. However, we concur with your view that beliefs (e.g., prediction) assessed in the BAQ involve the integration of external and internal information. Whilst we did not have data examining BAQ subscales (e.g., ‘predict body reactions’) and alexithymia, we have adjusted our discussion to highlight the BAQ's potential role in assessing both adaptive interoceptive accuracy and attention beliefs. (p. 44, L 1175-1182). *4. The authors propose to differentiate questionnaires based on their adaptivity aspects. Three comments: (1) Could the authors further discuss their definition of this criterion? (2) Could we categorize these questionnaires differently (e.g., based on the valence of sensations reported in the questionnaire)? (3) They should recommend future studies to test the validity of this classification into two categories (i.e., maladaptive vs. adaptive).*

Our responses to each of these great comments are as follows:

1. We have provided clarification on definitions of adaptive and maladaptive interoception in the Introduction (pp. 4-5, L 63-74), which contextualises later differentiation of adaptive and maladaptive forms of attention, accuracy, and interpretation, according to their impact on efficient physiological regulation (e.g., homeostasis/allostasis).
2. The categorisation of questionnaires based on the valence of sensations assessed is a thoughtful and considered suggestion from the Reviewer. While we understand the rationale behind this suggestion, we believe that categorising the questionnaires in this manner may not fully capture more specific constructs. For instance, several subscales from the MAIA contain items involving pleasant, unpleasant, and neutral sensations. It would be challenging to categorise these subscales according to sensation valence. However, the valence of sensations (e.g., pleasant, neutral, unpleasant) was considered in our differentiation of adaptive versus maladaptive interoceptive abilities, focusing on accuracy, attention, and interpretation. The differentiation of adaptive and maladaptive questionnaires is rooted in the emotional and regulatory outcomes associated with the interoceptive abilities they measure. While we acknowledge that the valence of sensations tacitly informed our differentiation (e.g., for measures such as the SAQ and BPQ Reactivity scales), we believe that the current categorisation provides a nuanced framework for understanding these measures, supported by emergent frameworks developed to improve the conceptualisation and measurement of interoception (e.g., Desmedt et al., 2023). We hope this explanation clarifies our position but acknowledge that the suggestion is a valuable one. We have adjusted our Discussion to clarify that our classification is merely one way to address the current inconsistencies in conceptualisation and measurement, suggesting that classification based on valence of sensations included in questionnaires may be another avenue worth pursuing (pp. 49-50, L 1329-1354).
3. We completely agree and have expanded on this in the Implications section (p. 48, L 1281-1284).

## **Reviewer #2**

*I commend the authors for what was clearly a massive undertaking. This area, as they clearly articulate, is a like a field of land-mines and they navigated it gracefully. I think, once cleaned up, this work will represent a clear contribution to research on interoception and alexithymia, as well as to research on emotional expertise more broadly. Really it is a tour-de-force. Please interpret my comments as constructive! I would ultimately love to see this published.*

We thank the Reviewer for their positive feedback on our endeavour, and greatly appreciate their considered feedback on aspects to improve the quality of our paper. We have provided our responses to each of their comments and suggestions below. *Overall comments:

1. I'll note first and foremost that I do not have personal experience conducting meta-analyses so I will leave it to another reviewer to comment on whether statistical analysis have been performed appropriately and rigorously. However I do want to applaud the careful approach the authors took to data disaggregation. I especially appreciated that the authors attempted to examine variability in effects across geographic regions.*

Many thanks to the reviewer for their comments regarding our methodological approaches! *2. I do want to acknowledge with full transparency that I had some difficulty following the logic of several sections. I recognize that it must be very difficult organizing so many theoretical perspectives, constructs, and ultimately, findings. I cant help but wonder if a bit more structure (e.g., headings) or sign-posting (e.g., in each results section we break down results based on effect size) would help the reader keep things straight in their mind. I also found several broken or grammatically messy sentences throughout, so I would encourage the authors to do another round of careful editing.*

We thank the author for their transparency in their experience reading the paper. We have done another round of editing and feel that the paper reads clearer. We also felt that the current headings are sufficient for containing results pertinent to each alexithymic outcome. We have therefore provided clearer signposting in the introductory paragraph of the ‘**The relationship between self-reported interoception and alexithymia’** results section, per the Reviewer’s suggestion (p. 23, L 602-603). We believe that this will enhance readability and keeping track of the results.

*Additional comments:

3. I think the authors need to be a bit more explicit about how their paper contributes above and beyond what's already been published by Trevisan et al. To be clear, I think this paper DOES contribute above and beyond what's already been published, but I think it wouldn't hurt to make this more explicit RIGHT AWAY (before overviewing Trevisan et al's specific findings). When you introduced the paper (which I hadn't read), my first thought was, "wait, did someone do this already?"*

We greatly appreciate this feedback. We have added a paragraph to describe how this study contributes above and beyond already published findings in the Introduction (p. 4, L 83-92).  *4. Related to point 2 above, in the introduction, the authors discuss (a) popular frameworks for organizing interoceptive CONSTRUCTS and (b) the various MEASURES used in interoceptive research with the goal (as I understand it) being to clarify how these two things are somewhat incoherent and poorly aligned. I wonder if using the language of "constructs" versus "measures" (i.e., a more explicit construct validity framework) would be helpful. As it is, the introduction mixes up discussions of both (a) and (b) in a way that was difficult to follow.*

We thank the Reviewer for this feedback and have updated the Introduction and Discussion sections to be more explicit in the discussion of constructs versus measures across the various frameworks. We have maintained some terminology (e.g., ‘factors’ and ‘subfactors’) according to the authors’ proposals per their frameworks.  *5. I recognize the need for acronyms but I would encourage the authors to go through and reduce their use of acronyms wherever possible. Particularly in the discussion, I found the prevalence of acronyms made it difficult to follow the narrate arc of each section. Acronyms should also be spelled out in footnotes for tables and figures. Especially given the complexity of your findings. Someone should be able to look at your figures and get the whole story.*

We thank the Reviewer for their openness in describing their experience reading the paper. Whilst many of the questionnaire acronyms are commonly employed in interoceptive research, you have highlighted something that also made us uncomfortable. We agree that the use of acronyms is extensive; however, the paper is already very long. Using the full names of the questionnaires would undoubtedly add to its length. Accordingly, we have decided to maintain the acronyms. We have ensured that an acronyms list and questionnaire descriptions has been provided as a Supporting Material (File S5, Table S4), and that all acronyms have been included in both the table footnotes and figures throughout the revised paper. Reviewer 3 suggested that the Discussion explains meta-analysis results rather than in terms of ‘positive’ and ‘negative’ relationships. The previous structuring of the discussion in this way was laden with acronyms. We have adjusted the Discussion to provide context for the questionnaire acronyms. Together, we feel that these compromises and changes will ensure the narrative arc can be followed.

*6. Please add a mean and standard deviation for participant ages if possible. 18-91 is a very broad age range and, given evidence that both self-report and task-based measures of interoception vary across the lifespan, this seems like a relevant detail.*

We thank the reviewer for their suggestion. We note that mean ages for samples have been provided in File S4 of the Supporting Information with the resubmission, which provides expanded characteristics of the included studies. *7. On line 331, can you clarify what you mean by "self-reported psychiatric diagnoses"? Are you distinguishing between formal and informal diagnosis here?*

We have clarified that the sample from this paper (Murphy et al., 2020, <https://journals.sagepub.com/doi/full/10.1177/1747021819879826>), per their reporting, included "participants who “reported an existing [psychiatric] condition” (Line 391). No formal diagnosis was undertaken by Murphy et al.  *8. Take this or leave it -- I personally think risk of bias assessments can go to the supplemental so that the main analyses about relationships among measures has a bit more breathing room.*

This is a great suggestion from the Reviewer, and we agree that breathing room would be particularly beneficial for readers of this paper. We have created a new Supporting file (File S3) containing the risk of bias assessment and moved the tables to this document (previously submitted as Tables S3 and S4 of Supporting Material).

*9. Line 527, you say "meta-analysis demonstrated a strong positive association for the ICQ and alexithymia..." and then on 530, "A strong positive effect was further observed between the ICQ and alexithymia..." I assume this is a mistake?*

Many thanks to the reviewer for identifying this typo! This has been amended to report results for the ICQ and **ISQ** in terms of their association with global alexithymia. (p. 24, L 653). *10. Throughout the paper, and in the discussion in particular, be careful when you use "interoceptive accuracy" since that will conjure up images of behavioral tasks for many readers given Garfinkel et als work. I would be very clear when you're referring to "task-based interoceptive accuracy" (e.g., on line 777?) vs self-reported interoceptive accuracy.*

We appreciate this advice and agree completely. We have amended this section to make the distinction between results concerning task-based/behavioural tests of accuracy and self-reports of accuracy more explicit. We have reviewed the paper to ensure that there is a strict specification of subjective vs. objective accuracy. *11. Line 835, you say, "DIF exhibited the highest magnitudes in associations with interoceptive self-report scales, which could be due scale construction and emotion generation processes. " I know this sentence has a typo, but on top of that I found it a bit confusing. Maybe consider something like, "which could be due to specific aspects of scale construction or to more general features of emotion generation processes. For example, ..."*

Thank you for this feedback. We have adjusted the discussion accordingly (p. 40, L 1046-1048). *13. On line 841 you write, "Additionally, theories of emotion propose that the interpretation of physiological sensations and changes plays an essential role in the generation of emotions, as they may initiate an affective state." The constructionist model (whose proponents you cite) would argue that "physiological sensations and changes" partially CONSTITUTE core affect. They would not argue that these sensations INITIATE affect. This is an important detail that these folks may be finicky about.*

Many thanks for this feedback. It is appreciated and, on balance, we agree that the original phrasing did not accurately reflect constructionist perspectives on emotion generation and experience. We have adjusted the Discussion to clarify that interoception as a constituent of conscious affect, which may contribute to emotional experiences (p. 41, L 1081). *14. I'm not sure i'm clear on your logic why the THISQ is a measure of "neutral interoceptive attention." You never really describe what you mean by "neutral."*

We have provided further reasoning for what the authors of THISQ mean by neutral in its subsection within Administered Measures (p. 21, L 541-543) and provided further evidence from their construct validity interpretation to support the classification of the THISQ as a measure of ‘neutral’ attention in the Discussion (p. 44, L 1184-1186). We hope this clarifies our reasoning.

*15. Would love to see a sentence or two on geographic variability given that you ran those analyses. Otherwise I would omit them from reporting and just say somewhere higher up in the results or methods that you ran those pre-registered subgroup analyses but have reported them in the supplement given small sample sizes. In general, I think any analyses that don't contribute to the main analyses (which are already complex) should be placed in the supplement.*

We would also have loved to have interpreted the influence of geographic variability and culture on the association! However, as the Reviewer rightly notes, the sample sizes were far too small within categories to detect significant differences with confidence. Per the Reviewer’s suggestion, we have removed these analyses from the main reporting and instructed the interested reader to review these at the online supplements hosted on the Open Science Framework (OSF). An overview of these results has also been added to OSF: <https://osf.io/ywg8z>.

## **Reviewer #3**

*Van Bael et al., conducted a thorough systematic review and several meta-analyses of currently used self-report interoceptive and alexithymia measures. Strengths of this research include the pre-registration and clear approach to assess the different measures used within the literature. I think that this study adds value to the current literature as it adds clarity to a field which is limited by its lack of consistency in definitions and measures. Further, the study is well-written. Therefore, my comments are minor, and I think that this will be a useful work to guide future researchers interested in investigating interoception and alexithymia.*

Many thanks to the Reviewer for their positive feedback and suggestions to enhance the quality of our paper. We have provided responses to each below. *• In the eligibility criteria, I think that it should be noted that clinical disorders were considered (i.e., they were not excluded).*

Thank you for this suggestion. We have added this to the eligibility criteria.

*• Given that the meta-analyses produces a lot of results, a summary figure/s would also enhance this study and help readers to understand the overall picture. This could be for example in the form of a spider plot or heat map showing the negative and positive correlations between each alexithymia domain and the interoceptive measures.*

This is a great suggestion from the Reviewer. We have included a heatmap figure, which provides an illustration of the correlations between alexithymia domains and various interoceptive measures (Fig 5 in revised manuscript).

*• Revise manuscript for repetition in results to increase conciseness. For example,
-Regarding the primary meta-analyses: can authors provide a workflow or diagram which summarises how many meta-analyses were conducted across the alexithymia scales? Instead of repeating this at the beginning of each subheading of the results (i.e., line 521, 582, 671).
-Lines: 568-571; 620-622; 695-697: regarding subgroup analyses. This sentence is repeated throughout the results but should be placed in the statistical analysis section of the methods and stated once.*

We thank the reviewer for this feedback. We have provided a workflow to summarise the number of primary and secondary meta-analyses conducted across each alexithymic outcome and subgroup factor (now Fig 4). In accordance with Reviewer 2’s feedback, we removed the subgroup analysis from the main reporting in the paper, due to the small sample sizes. Further Supporting information has been provided for interested readers to access secondary meta-analysis/subgroup analysis results via the online registry links (OSF) and <https://osf.io/ywg8z>.

*- Broadly, I think that references to the statistical thresholds and effect size cut-offs could be moved to the methods (Line 510-518), together with the information about the sub-group analyses.*

We thank the Reviewer for this comment. Statistical thresholds and effect size cutoffs has been moved to the Methods. Following feedback from Reviewer 2, results for the subgroup analyses are now provided on OSF by accessing the relevant links. An overview has been provided at <https://osf.io/ywg8z>.

*• In discussion, avoid using “positive” or negative” association and instead explain the relationship “i.e., greater alexithymia was associated with higher levels of interoceptive confusion” Line 754.*

We appreciate this feedback from the Reviewer. We have adjusted the Discussion to explain the observed relationships.

*• Some measures are more popular or well-known than others (i.e., TAS-20, MAIA) and therefore may have had more weight in each meta-analysis. Authors should mention how this might have influenced their results, as some frequencies of use = 1 (i.e., Figure 2), so I would think that these results need to be interpreted with some caution.*

We agree and have added this to the Limitations section (p. 50, L 1335-1357). *Minor comments:
Line 174: wording “as meta-analysis” change for “meta-analytic approaches should enable the quantification”*

This has been changed (L 210). *Line 245: wording “meta-analysed*

This has been amended to “Separate meta-analyses were conducted according to specific interoceptive scales…” (L 292-293) *Line 281: Typo “Supplemental”*

‘Supplemental’ has been replaced with ‘online supplements. (L 333) *Line 380: Move the section of excluded scales to end of section to improve flow.*

This has been moved to the end of the section. (L 550) *Line 714: Avoid using the word “forced” due to negative connotations. Replace with more neutral word like “used” or “employed”*

Forced has been replaced with employed. (L 875) *Line 1076-1078: Link results of current study to this sentence as it is currently not clear that using appropriate measures of interoception and alexithymia will likely improve therapeutic outcomes.*

We have linked our meta-analysis results to these implications (p. 49, L 1311).  *Figure 1: check figure quality*

We have updated the quality for all figures.
