# Supplementary material for: A systematic review and meta-analysis of the relationship between subjective interoception and alexithymia: Implications for construct definitions and measurement
Source: PLoS One. 2024 Nov 7;19(11):e0310411. doi: 10.1371/journal.pone.0310411 (PMC11542822; doi:10.1371/journal.pone.0310411)
Supplement: Attachment — Submitted filename: Response to Reviewers.docx [file pone.0310411.s012.docx]

# **Reviewer #1**

I would like to thank the authors for their exhaustive answers. I, nevertheless, have some remaining comments.

*1. The methodology employed to assess inter-rater reliability is overall good, but also reveals some areas for potential improvement. For the title and abstract screening phase, the reported kappa values (κ = 0.10-0.39) indicate slight to fair agreement following the most liberal guidelines (Landis & Koch, 1977), but poor (Fleiss, 1981; Altman, 1991) following more conservative ones. This means that the replicability of this selection phase is not optimal and should be acknowledged in the limitations. For the extraction phase, the percentage agreement shows a high level of reliability (84.4 to 90.6%), which is commendable, but the reliance on percentage agreement alone, without considering chance agreement, may overestimate true reliability. This should also be acknowledged in the limitations.*

Many thanks to the reviewer for this observation. We agree that these aspects of inter-rater reliability were suboptimal and have acknowledged this in the limitations of the revised manuscript (L 1212-1215).

*2. Although I have explained that the THISQ and the BAQ are not measures of interoceptive attention, the authors stand by their interpretation based on the following arguments: (1) The authors of the THISQ (Vlemincx et al., 2023) note that THISQ scores correlated moderately with other measures of non-emotional interoceptive attention, such as the BAQ and the Noticing scale of the MAIA; (2) The BAQ is currently classified as a self-report of interoceptive attention in the literature (e.g., Trevisan et al. 2020; Vlemincx et al. 2023), as noticing and frequency of awareness involves orienting attention to salient interoceptive signals (Mehling, 2012). Here are my answers: (1) the authors of the THISQ wrote: “Self-reported interoception as assessed by the THISQ is defined as the self-reported awareness or observation of these sensations, assessed by the rate at which persons notice or feel (changes in) these internal bodily sensations. By assessing the ‘noticing’ and ‘feeling’ of sensations, we excluded interoceptive features such as discrimination, accuracy and attention, to avoid specific emotional or cognitive interpretations of sensations (similar to the BAQ and BPQ)” (p.1238). They also wrote: “… the THISQ assesses self-reported interoception... However, it does not explicitly assess interoceptive accuracy, nor interoceptive attention… The THISQ items are not subjective evaluations of whether one pays attention to these sensations, nor of whether one perceives these sensations accurately.” (p.1249) A moderate correlation does not support the conclusion that two measures evaluate the same construct. It is expected that a measure of attention correlates with a measure of awareness, as more attention should lead to more awareness; the two constructs are related but distinct. Put differently, while attention is necessary for individuals to notice and be aware of their sensations, it is not sufficient. (2) For the BAQ, it is more debatable as the original authors have themselves qualified this questionnaire as a measure of interoceptive attention. Again, however, although I agree that attention would help to better notice or predict bodily sensations, I do not agree that the BAQ is a direct measure of interoceptive attention given that no item refers to attention per se.*

We greatly appreciate these comments regarding the THISQ. On balance, classification of this questionnaire is indeed challenging—particularly in the context of its overall very weak association with alexithymia. As the Reviewer rightfully notes (and we thank them for including the excerpt from the original article), Vlemincx et al. (2023) regard the THISQ as measuring “the self-reported awareness or observation of these sensations, assessed by the rate at which persons notice or feel (changes in) these internal bodily sensations.*”* We personally feel that classification of the THISQ as a measure of interoceptive awareness is not suitable, given the issues with this umbrella term in the literature, described in the ‘Interoceptive constructs and measurement’ section of the Discussion (p. 48).

In their previous comments regarding the THISQ, the Reviewer proposed that “neutral interoceptive detection would be more relevant” for classification of the THISQ, which is more specific and encouraged in the manuscript. Due to the valid conceptual issues raised by the Reviewer, we made the decision to relabel ‘interoceptive accuracy’ to ‘interoceptive sensing’. This decision was in accordance with how Desmedt et al. (2023, pp. 14-15) have defined this construct: “the sense of internal signals by the nervous system across conscious and nonconscious levels…Interoceptive sensing may be underpinned by interoceptive detection (called “interoceptive accuracy” by Garfinkel et al., 2015), interoceptive magnitude (Khalsa et al., 2018), and interoceptive localization (called “interoceptive discrimination” by Khalsa et al., 2018)”. We feel that this re-classification, whilst broader in terms of the included and applicable constructs, encompasses the Reviewer’s perspectives on classification of the THISQ.

Given the amendment of (adaptive and maladaptive) ‘interoceptive accuracy’ initially proposed to ‘interoceptive sensing’, this necessitated review, and in some instances, reclassification of other questionnaires. One such scale was the Noticing scale from the MAIA, currently regarded as an aspect of interoceptive attention in the literature. We agreed with the points made in the Reviewer’s third comment regarding underlying subfactors of interoceptive attention. Noticing MAIA seem more in line with the proposed construct of ‘interoceptive sensing’, as the items pertain to the basic detection of comfortable, neutral, and uncomfortable sensations, which may implicitly involve and be strengthened by interoceptive attention, but is ultimately not dependent on this process—nor do items explicitly refer to attentional control toward sensations.

Classification of the BAQ is particularly troublesome, given the classification of it as both a measure of (potentially maladaptive) interoceptive attention characterised by somatisation in the literature (e.g., Trevisan et al., 2021), and within the authors’ description of the scale (Shields et al., 1989). It is unfortunate that meta-analysis could not be conducted on the four subscales comprising the BAQ (i.e., Note Response/Changes in Body Process”: ability to read one's own body reaction to food, fatigue, weather, and changing in energy levels; “Predict Body Reaction”: ability to predict one's own body state from actual body signal; “Sleep-Wake Cycle”: bodily signals related to circadian rhythm; “Onset of Illness”: ability to recognise illnesses signals). Given that the BAQ has remained a point of concern for the Reviewer, we recognise that our initial reply to their comment did not adequately address this. Accordingly, qualitative item analysis was conducted to determine what subjective interoceptive aspects/beliefs are measured by BAQ items. We identified that the items covered various interoceptive domains, including detection (noticing subtle changes in bodily signals), accuracy (correctly identifying interoceptive signals), prediction (forecasting future bodily states based on current sensations and environmental factors), and differentiation (distinguishing between different bodily states). Following this review, we agree with the Reviewer—the BAQ should not be conceptualised as a self-report measure of (adaptive) interoceptive attention. We have therefore re-classified the BAQ has a measure of adaptive interoceptive sensing but note that the scale also contains measurement of predictive ability (e.g., onset of illness, predict body reaction). We feel that this these subjective beliefs are possibly in line with ‘interoceptive memory’, as conceptualised by Desmedt et al. (2023, p.14), “involving any memory process related to internal signals”. Such capacities can potentially be understood within predictive accounts of interoception, as the ability to anticipate and prepare for future body states and needs is inherently based on past experiences and current interoceptive and environmental cues. Past experiences and memory plays a key role in this process, as it enables consolidation and encoding, enabling individuals to predict internal bodily sensations and needs based on their prior experiences deemed relevant for homeostasis and allostasis (e.g., Barrett et al. 2016, <https://www.affective-science.org/pubs/2016/active-inference-allostasis-ptb.pdf>).

Accordingly, the manuscript has been adjusted to reflect these substantiative changes, which are peppered throughout the Abstract, Introduction, and primarily, the Discussion section to reflect this revision, particularly in the following sections: Key differences in interoceptive self-reports, Implications, Conclusion.

*3. This debate about the construct (i.e., whether or not it is interoceptive attention) measured by certain questionnaires is not without consequences. Indeed, the authors conclude that alexithymia is associated with more interoceptive attention. I do not agree. Questionnaires relevant to interoceptive attention are the Interoceptive Attention Scale (IATS) and the subscales MAIA-SR and MAIA-AR. Each of these (sub-)scale, however, measures different subcomponents of interoceptive attention. While the IATS evaluates whether individuals pay their attention “most of the time” towards bodily states indicating homeostatic disturbances (or hypervigilance towards body needs), the MAIA subscales evaluate the ability to sustain and control attention to body sensations and the ability to regulate distress through bodily attention. Based on the results, and to simplify, we could thus conclude that alexithymia is associated with more maladaptive interoceptive attention and less adaptive interoceptive attention. This conclusion further demonstrates that “interoceptive attention” covers different subcomponents and should not be uniformly assigned to multiple questionnaires (THISQ, IATS, BAQ, and MAIA subscales), especially when their items do not refer to attention.*

We thank the Reviewer for again sharing their valuable insights and perspectives. Interoceptive attention is a particularly nebulous construct, given that various definitions and categorisations of what constitutes attention vastly differ across the literature (e.g., Khalsa et al., 2018, Desmedt et al., 2018, Murphy et al., 2019, Suksasikp & Garfinkel, 2022). Firstly, we wish to confirm that we concluded alexithymia is associated with maladaptive interoceptive attention, per the associations with IATS. Although the manuscript alluded to this scale tapping into hypervigilance, we have reiterated this in the revised discussion. Moreover, we initially concluded that lower alexithymia is associated with more adaptive interoceptive attentional beliefs. In the Discussion section, we have provided a brief clarification following discussion of the ‘adaptive’ aspects in discussion of the associations with global alexithymia to advise that the converse applies: less adaptive interoceptive beliefs are associated with higher alexithymia (L 846-848).

Subsequent to our revision of accuracy to ‘interoceptive sensing’, we note that MAIA-Noticing and BAQ are no longer classified as measures of interoceptive attention in the revised manuscript. Additionally, MAIA-BL has been re-classified as a measure of adaptive interoceptive interpretation, as items regard the use and interpretation of bodily signals to guide decision-making, making sense of or resolving emotional distress, and belief that the body serves as a valuable source of emotional information. The revised classification of the interoceptive attention construct now includes three adaptive measures (MAIA-AR, MAIA-SR, MAIA-ND), and one maladaptive measure (IATS). We retained MAIA-ND as a measure of adaptive interoceptive attention, as Desmedt et al. (2023) have previously contended that this measure is an aspect of attention, coupled with reverse-scoring of items suggesting that higher ND scores are akin to attending to and accepting sensations of pain and discomfort, relative to distraction and avoidance—aspects underpinning maladaptive interoceptive attention styles (Khalsa et al., 2018). These changes have been made to the manuscript, and peppered throughout the Discussion section.

We are inclined to concur with Desmedt et al., in that these subjective beliefs, assessable via self-report, could broadly considered as measures of interoceptive attention. However, as Desmedt and the Reviewer note, these measures should not be regarded as tapping into the same aspect of attention (or sensing, interpretation, and memory) until evidence for convergence is established. This is a notion that we completely agreed with in the original drafting and first revision. However, it is clear from the Reviewer’s comments that this conclusion was not explicitly phrased. We have adjusted the Discussion, specifically the Implications section, to express this (L 1157-1161), and again, thank the Reviewer for highlighting this important contention.

We have provided an overview of previous and current classifications at the end of this document.

*Besides these concerns, the manuscript is excellent. Once my comments have been further addressed, I recommend its publication.*

Many thanks to the Reviewer for their insights during this process. We believe that we have adequately considered and addressed their concerns. The overall comments have enabled us to greatly refine the proposed construct validity framework for interoceptive self-report scales, resulting in clearer and more specific classifications.

# Reviewer #2

*Thank you to the authors for their thorough response to my review. I believe the paper is both clearer and more concise. I can happily recommend this paper for publication. I look forward to citing it.*

Many thanks to the Reviewer! Their comments have greatly improved the clarity of the paper and further in reducing confusion regarding the THISQ.

*A few remaining comments below:

1.Regarding the use of “neutral” – I still struggle a bit with this. I think it’s valid to relay that the authors of the THISQ used the term, but I’m not sure the term is useful in the context of your larger discussion. Specifically, it’s not clear to me that-on-the-whole, people have great access to physiological sensations that are “neutral.” Given the neurobiology of the system and available data, it seems moreso that most “typical” physiological phenomena are not experienced within conscious awareness. I’m also thinking of the newer ESM paper by Geoff Bird’s group, demonstrating that most often when people report attending to bodily sensations, they also report small increases in negative affect. All-in-all, I can’t imagine I’m the only one that will get distracted by the term “neutral.” Perhaps the distinction is more-so “self-reported perception of body sensations at rest (e.g., normal breathing) versus at times of physiology strain or exertion (e.g., dyspnea)?”*

Many thanks to the Reviewer for alerting us to the ESM paper! We have incorporated those findings into discussion of the IATS (L 983), as this is the scale the study utilised to operationalise interoceptive attention. We have classified the IATS as a measure of maladaptive interoceptive attention, given that its items are indicative of hypervigilance and homeostatic disturbance, which may explain the observed association between increased attention in daily life with increased negative valence and fatigue.

In addressing the comments regarding our conceptualisation of the THISQ as a measure of neutral interoceptive attention from Reviewer 1, we have re-classified the THISQ as a potential measure of adaptive interoceptive sensing. Interoceptive sensing has replaced the interoceptive accuracy construct previously proposed. This domain, in accordance with Desmedt et al.’s (2023, pp. 14-15) conceptualisation of the construct, involves: “the sense of internal signals by the nervous system across conscious and nonconscious levels…Interoceptive sensing may be underpinned by interoceptive detection (called “interoceptive accuracy” by Garfinkel et al., 2015), interoceptive magnitude (Khalsa et al., 2018), and interoceptive localization (called “interoceptive discrimination” by Khalsa et al., 2018)”. The THISQ has been re-classified as a measure of interoceptive sensing, as items arguably involve the capacity to detect subtle changes at rest and following exertion. We have removed the term ‘neutral’ in discussing this measure and have termed this scale as adaptive, given the positive associations with the BAQ and MAIA-Noticing scale, which the THISQ moderately correlates with (L 963). Moreover, these two scales, in meta-analysis showed negative associations with alexithymic outcomes, suggesting that they capture more adaptive sensing beliefs. We feel that this updated classification should reduce some of the concerns that the Reviewer has regarding the use of the term ‘neutral’.

*2.Lines 95-96, I would recommend using a different phrasing besides “heartbeat tracking tasks”. This phrasing is often used to describe heartbeat counting or tapping tasks. For both these tasks, I’ve seen disagreement in the literature about whether they belong in the “accuracy” or “sensibility” categories. I would maybe recommend instead citing “heartbeat detection or discrimination tasks.”*

Per the reviewer’s suggestion, this has been adjusted in the revised manuscript.

*3.Lines 104-107 (and elsewhere in the manuscript)—I would place the different constructs in quotations to clarify that these are terms introduced by the meta-analyzed work, and not terms that are necessarily endorsed by the authors. This would also help to differentiate when you’re engaging in “review” verses “inference.”*

This is a great suggestion from the Reviewer. We have placed the different and previously delineated constructs in inverted commas throughout the manuscript.

*4.Very nit-picky, but on 114-115, I may also recommend including “sickness” (e.g., chills or fever) or “injury” (e.g., bruising) as bodily signals represented in common self-report measures of interoceptive ability. I say this because sickness in particular, unlike hunger, thirst, fatigue, or cardiovascular sensations are not typically discussed in work on interoception (and is not objectively measured by behavioral tasks). This is one place where I think refinement of our construct is vital.*

This nit-picking is greatly appreciated. We agree that illness and injury are often overlooked in considering the array of interoceptive sensations. We have adjusted our introduction to include illness and injury as assessable aspects via self-report.

*5.Line 565-566, please include a parenthetical indicating where these “weak associations” can be found (e.g., “see Table X).*

This has been added.

# Reviewer #3

*The authors carefully addressed my concerns and revised their manuscript thoroughly. Well done to the authors on this solid piece of work.*

Many thanks to the Reviewer! Their previous comments greatly contributed to the quality of the current manuscript.

# Classification of Interoceptive Self-Report Scales

| Scale | Previous Classification | Revised Classification |
| --- | --- | --- |
| BAQ | Adaptive Interoceptive Attention | Adaptive Interoceptive Sensing and Memory |
| BPQ-Reactivity (sub, supra) | Maladaptive Interoceptive Interpretation | Maladaptive Interoceptive Interpretation |
| EDI-IAw | Maladaptive Interoceptive Accuracy | Maladaptive Interoceptive Sensing |
| IAS | Adaptive Interoceptive Accuracy | Adaptive Interoceptive Sensing |
| IATS | Maladaptive Interoceptive Attention | Maladaptive Interoceptive Attention |
| ICQ | Maladaptive Interoceptive Accuracy | Maladaptive Interoceptive Sensing |
| MAIA-AR | Adaptive Interoceptive Attention | Adaptive Interoceptive Attention |
| MAIA-BL | Adaptive Interoceptive Attention | Adaptive Interoceptive Interpretation |
| MAIA-EA | Adaptive Interoceptive Interpretation | Adaptive Interoceptive Interpretation |
| MAIA-ND | Adaptive Interoceptive Attention | Adaptive Interoceptive Attention |
| MAIA-Noticing | Adaptive Interoceptive Attention | Adaptive Interoceptive Sensing |
| MAIA-NW | Adaptive Interoceptive Interpretation | Adaptive Interoceptive Interpretation |
| MAIA-SR | Adaptive Interoceptive Attention | Adaptive Interoceptive Attention |
| MAIA-Trusting | Adaptive Interoceptive Interpretation | Adaptive Interoceptive Interpretation |
| SAQ | Maladaptive Interoceptive Interpretation | Maladaptive Interoceptive Interpretation |
| THISQ | Adaptive Interoceptive Attention | Adaptive Interoceptive Sensing |
